# Does physical activity really improve anxiety and depression in overweight or obese children and adolescents? A systematic review and meta-analysis

**DOI:** 10.1186/s12888-025-07761-9

**Published:** 2026-01-16

**Authors:** Jie Men, Yuxi Zhang, Simin Wu, Pengbo Wang, Zhengyang Yu, Guoyu Zhu, Jingwen Wang, Weiqi An, Zhaowei Li, Penghong Liu

**Affiliations:** 1 Shanxi College of Medicine, Fenyang, 032200 China; 2 Key Discipline of Physiology, Shanxi College of Medicine, Fenyang, 032200 China; 3 The First Clinical Hospital of Shanxi Medical University, Taiyuan, 030001 China

**Keywords:** Obese, Overweight, Children, Adolescents, Physical activity, Anxiety, Depression

## Abstract

**Objective:**

To evaluate the effects of physical activity (PA) on anxiety, depression, self-esteem, and self-worth in overweight/obese children and adolescents.

**Methods:**

A systematic search of six databases was conducted from inception to March 1, 2025. Randomized controlled trials (RCTs) examining the effects of PA on mental health outcomes in children and adolescents (aged 5–19 years) with overweight/obesity were included.

**Results:**

Nineteen RCTs (1,795 participants) were analyzed. PA was associated with improvements in anxiety (SMD = -0.98, 95% CI -1.90 to -0.05, *P* = 0.04), depression (SMD = -0.15, 95% CI -0.25 to -0.05, *P* = 0.005), self-esteem (SMD = 0.19, 95% CI 0.03 to 0.35, *P* = 0.02), and self-worth (SMD = 0.34, 95% CI 0.19 to 0.49, *P* = 0.0002). However, the evidence for anxiety was of low certainty, and the improvements in depression and self-esteem were modest. The effects were also modulated by age, obesity level, and race.

**Conclusion:**

PA was associated with small to modest improvements in mental health outcomes among young people. While PA may reduce anxiety, the evidence is uncertain, and the observed effects on depression and self-esteem appear small and of modest clinical relevance, supporting PA as an adjunctive mental health strategy.

**Clinical trial number:**

Not applicable.

**Supplementary Information:**

The online version contains supplementary material available at 10.1186/s12888-025-07761-9.

## Introduction

The comorbidity of overweight or obesity and depression among children and adolescents has emerged as a significant public health challenge spanning the entire life cycle. This condition leads to adverse health outcomes with multidimensional cumulative effects, encompassing physiological, psychological, and social functioning [1]. The World Health Organization (WHO) 2024 report reveals that the global prevalence rate of depression among children and adolescents stands at 6.2% [2]. Notably, this figure escalates significantly to 26.7% in the context of overweight and obesity conditions [3]. Furthermore, anxiety is a prevalent mental health condition in this population [4], with 25% to 50% of depressed youth presenting comorbid anxiety symptoms. Studies have demonstrated that both obesity and depression in childhood and adolescence serve as potent predictors of negative long-term outcomes [5, 6]. Of particular concern is the fact that over 50% of obese children [7] and up to 67% of those with depressive symptoms [8] will continue to experience these issues into adulthood, significantly heightening their risk of developing type 2 diabetes, hypertension, cardiovascular diseases, and cancer [9]. Moreover, these individuals face an increased propensity for substance abuse, psychiatric disorders [10] and severe consequences including self-harm and suicidal behavior [8]. The all-cause mortality rate in this population is six times higher than that of healthy individuals [11]. Therefore, mitigating the adverse effects of the “obesity-depression” bidirectional relationship highlights the critical importance of early intervention measures.

Clinical guidelines, including those from the American Psychiatric Association (APA) [12] and Psychological Interventions Implementation Manual [13], recommend pharmacological and psychological treatments as first-line interventions for children and adolescents. Nonetheless, their implementation in clinical practice is fraught with challenges: drug therapies may induce side effects such as gastrointestinal disturbances, behavioral abnormalities, and even increased suicidal ideation [14, 15], whereas psychological interventions are often hampered by high costs and limited accessibility, with only 36% of affected individuals receiving treatment [16]. Particularly for children and adolescents with comorbid overweight/obesity and depression or anxiety, traditional therapies’ neglect of weight management may exacerbate the risk of metabolic disorders. By comparison, physical activity (PA), as a non-pharmacological intervention, offers dual benefits for psychological and metabolic well-being through its effects on neurobiological pathways (e.g., enhancing neuroplasticity and mitigating inflammation) and psychosocial mechanisms (e.g., elevating self-esteem, fostering social support, and regulating mood). Its efficacy has been recognized by leading guidelines, including those from the WHO and NICE [17]. Nonetheless, the current recommendation for its role as an adjunctive therapy might be excessively cautious [18], a finding derived from prior meta-analytic evidence [8, 18, 19]. In addition, unlike conventional treatment approaches, PA is not associated with serious adverse effects or therapeutic biases, and it demonstrates remarkable effectiveness in improving outcomes related to overweight or obesity [20]. These positive effects highlight the unique advantage of PA in simultaneously addressing the dual health challenges of obesity and anxiety-depression. Furthermore, the flexibility of PA (e.g., home-based exercises, school programs) effectively overcomes the inherent accessibility limitations of traditional therapeutic approaches.

While prior meta-analyses have sought to systematically assess the effects of PA on depressive symptoms among children and adolescents, only a single study by Lan Chen et al. [21] has specifically targeted the overweight or obese pediatric population. Nevertheless, its findings should be interpreted with caution due to several methodological limitations, including the inclusion of 40% non-RCTs, one study that did not focus on PA interventions, and the presence of some healthy-weight participants. These factors may undermine the reliability and precision of the conclusions drawn.

The aim of this meta-analysis was to assess the effects of PA on anxiety and depression in overweight/obese children and adolescents, incorporating self-esteem and self-worth as established predictors of depression [22]. Additionally, we extended prior research by exploring how age, obesity degree, and racial factors may influence the results, offering an evidence-based foundation to inform clinical or family health management practices and the formulation of exercise guidelines and recommendations.

## Methods

### Protocol registration

This study was registered with PROSPERO (CRD42024593434) and conducted under preferred reporting programs that follow the Cochrane Collaboration Network recommendations and guidelines and the Preferred Reporting Items for Systematic Reviews and Meta-Analyses (PRISMA) reporting guidelines [23].

### Search strategies and eligibility criteria

In this study, we comprehensively searched six Chinese and English databases to ensure both breadth and depth of literature coverage. PubMed and Embase, as core international biomedical resources, provided high-quality original research; Web of Science and Cochrane Library supplemented authoritative information from interdisciplinary journals and systematic reviews. Given that China bears one of the countries bearing the heaviest global burden of childhood overweight and obesity, a substantial number of Chinese studies are published exclusively in CNKI and Wanfang databases. Including these two databases helped reduce language and publication bias. As studies published in other non-English languages are largely indexed in major international databases, no additional searches of language-specific databases were conducted. Through a comprehensive search of the aforementioned databases, original research in Chinese and English published up to March 1, 2025, was selected. Manual searches of reference lists from previously published meta-analyses and reviews were performed to identify any potentially missed studies. No new human participants were recruited for this meta-analysis; all analyses were based solely on published studies and de-identified, aggregated data. Guided by the PICOS framework, the search strategy incorporated terms such as “overweight,” “obesity,” “children,” “adolescents,” “physical activity,” “mental health,” “anxiety,” “depression,” “self-esteem,” and “self-worth.” For example, to capture the target population in PubMed, we used the following terms: “adolescent“[MeSH Terms] OR “child“[Title/Abstract] OR “young“[Title/Abstract] OR “boy“[Title/Abstract] OR “girl“[Title/Abstract] OR “teen“[Title/Abstract] OR “kid“[Title/Abstract] OR “school age“[Title/Abstract] OR “schoolchild“[Title/Abstract] OR “youth“[Title/Abstract] OR “juvenile“[Title/Abstract]. The Meta-analysis timeline, search process, and full Boolean search strings are listed in Appendix File 1–2. To ensure objectivity in the study selection and quality assessment processes, the roles of the researchers are replaced with using coded identifiers (R1, R2, and R3) in this section. The search strategies and results were developed and screened by bilingual researchers (R1 and R2) to ensure the accurate interpretation of both Chinese and English studies. These were then independently verified by a third researcher (R3) to guarantee accuracy and reproducibility. Inter-rater agreement for the screening process was quantified using Cohen’s kappa coefficient. 0.01–0.20, slight agreement; 0.21–0.40, fair agreement; 0.41–0.60, moderate agreement; 0.61–0.80, substantial agreement; and 0.81-1.00, almost perfect agreement. Any disagreements between the two researchers (R1 and R2) were resolved through discussion, and if consensus could not be reached, the issue was adjudicated by the third researcher (R3).

This study included children and adolescents aged 5–19 years with overweight or obesity. The age range was defined according to the WHO. Overweight and obesity were determined based on international or national pediatric BMI criteria, including the CDC age-specific BMI percentiles [24], WHO zBMI scores [25], IOTF age- and sex-specific BMI cut-offs [26, 27], and authoritative national standards applicable in the study setting.

Inclusion Criteria: (1) Children and adolescents aged 5–19 years [28] who were classified as overweight or obese (based on child/adolescent BMI standards) [29], (2) Intervention: The intervention was centered on PA as the core component. PA could be delivered alone or in combination with other lifestyle components (e.g., dietary education, psychological support), (3) Control Measures: The control groups did not receive any PA-based intervention. As long as no structured PA intervention was provided, they could receive other forms of intervention, such as usual care, health education, dietary counseling, or psychological support. (4) Outcome Measures: Anxiety, depression, self-esteem, and self-worth were assessed using standardized psychometric tools (e.g., Beck Depression Inventory [BDI] or Harter Global Self-Esteem Subscale), (5) RCTs.

Exclusion Criteria: (1) Participants: Professional athletes or individuals with significant contraindications to exercise or infectious diseases, (2) Intervention: Studies that involved games relying solely on static activities (e.g., meditation) or traditional gaming devices (e.g., controllers, keyboards) were excluded, (3) Control Measures: Control groups that involved any form of PA intervention were excluded, (4) Outcome Measures: Studies with incomplete data or those not utilizing standardized psychometric tools, (5) Study Design: Cross-sectional studies, conference abstracts, non-RCTs, and review articles.

### Outcome

This study selected anxiety, depression, self-esteem, and self-worth as outcome measures based on the specific psychological risks faced by children and adolescents with overweight/obesity. The primary outcome was assessed as the mean change in anxiety and depression scores from baseline to the end of the intervention. For studies employing multiple scales, observer-rated scales were prioritized over self-reported ones. Given the variability in anxiety/depression scales across studies, the results were expressed as mean differences to minimize heterogeneity. It should be noted that in this study, self-esteem and self-worth were treated as distinct psychological constructs. Self-esteem refers to an individual’s overall self-evaluation and confidence, while self-worth pertains to the perception of competence in specific domains. Both were assessed with different standardized instruments and were analyzed as independent outcome variables.

### Data extraction

The retrieved literature was imported into EndNote X9 for deduplication. Two independent bilingual researchers (R1 and R2) screened titles and abstracts to exclude studies unrelated to the research focus. Full texts of potentially relevant studies were then obtained for detailed assessment. A standardized data extraction form was developed to capture essential study characteristics. The form included details such as the first author, publication year, author nationality, article title, and journal. It also documented participant demographics (e.g., age, gender, sample size), obesity subtype, ethnicity, adverse events (including statistics and causes), and specific outcome measures. Furthermore, the trial registration status (registered, unregistered, or discrepant) was recorded for use in the risk of bias assessment (see Appendix File 3). To ensure the statistical independence of effect sizes in the subsequent meta-analysis, studies with multiple intervention arms sharing a single control group were handled by splitting the control group sample size to construct independent comparisons, and outcome data were consistently extracted from the assessment create closest to the end of the intervention. Data extraction was performed independently by the two researchers (R1 and R2), followed by cross-verification. Discrepancies between the two researchers (R1 and R2) were resolved by jointly reviewing the full texts to reach a consensus. If consensus could not be reached, a third researcher (R3) was consulted for further discussion and resolution. For studies with missing or incomplete data, the corresponding authors were contacted via email to obtain supplementary information. Studies lacking essential quantitative data (e.g., standard deviations or between-group comparisons) were excluded from the quantitative synthesis.

### Risk of bias assessment

The Cochrane Risk of Bias Tool 2.0 [30] was used to evaluate the quality of RCTs, including five different areas: (1) bias arising from the randomization process; (2) bias due to deviations from intended interventions; (3) bias due to missing outcome data; (4) bias in measurement of the outcome; and (5) bias in selection of the reported result. Two researchers (R1 and R2) independently assessed them according to three risk levels (low risk, high risk, and some concerns), with inter-rater agreement quantified using Cohen’s kappa coefficient (κ = 0.82, indicating substantial agreement). Any discrepancies identified during the assessment were resolved through discussion between the two researchers (R1 and R2). If a consensus could not be reached, a third researcher (R3) was consulted for arbitration.

### Statistical analyses

To synthesize data from studies using different rating scales, we used Hedges’ g (a bias-corrected standardized mean difference, SMD) as the effect size metric [31]. All effect sizes were calculated using pre-to-post change scores for both the intervention and control groups. All effect sizes were computed from pre–post change scores in the intervention and control groups (ΔMean = Mean_{post} − Mean_{pre}). When SD of the change score was not reported, we imputed it using SD_Δ = √(SD_{pre}² + SD_{post}² − 2r·SD_{pre}·SD_{post}), assuming a pre–post correlation of *r* = 0.50; in accordance with the recommendations of the Cochrane Handbook for Systematic Reviews of Interventions [32] and previous methodological studies that commonly assume a moderate baseline–follow-up correlation when imputing the SD of change [33]. Sensitivity analyses used *r* = 0.30 and 0.70. The change-score SMD was calculated as $\:({\Delta\:}Mea{n}_{I}-{\Delta\:}Mea{n}_{C})/S{D}_{pooled,{\Delta\:}}$and multiplied by Hedges’ small-sample correction factor. Effect sizes are reported as Hedges’ g with 95% confidence intervals. Absolute SMD (Hedges’ g) values of 0.2, 0.5, and 0.8 were interpreted as small, medium, and large effects, respectively [34]. Directionality was outcome-specific: negative SMDs indicated improvements (reductions) in anxiety and depressive symptoms, whereas positive SMDs indicated improvements (increases) in self-esteem and self-worth.

Heterogeneity was quantified using the I² statistic with 95% confidence intervals and the between-study variance (τ²). Given that the included studies differed in population characteristics (e.g., country, health status, gender distribution) and used various psychometric instruments with different scoring properties and cultural adaptations, we anticipated substantial methodological and clinical diversity. Thus, a random-effects meta-analysis was applied for all outcomes to reflect the assumption that true effects vary across studies. Between-study variance (τ²) was estimated using the restricted maximum likelihood (REML) estimator. Pooled effect sizes and their 95% confidence intervals (CIs) were obtained using the Hartung-Knapp-Sidik-Jonkman adjustment, which accounts for uncertainty in the estimation of τ². Additionally, we computed 95% prediction intervals to express the expected range of true effects in a new study conducted under similar conditions.

We conducted subgroup analyses based on age, degree of obesity, and race to examine the potential moderating effects of these factors on the magnitude of PA effects. Age was categorized into children (5–12 years) and adolescents (13–19 years) according to Erikson’s theory of psychosocial development [28]. Obesity status was determined based on international or national pediatric BMI standards, and classified as overweight (BMI ≥ 85th and < 95th percentile, or zBMI > + 1 SD) and obesity (BMI ≥ 95th percentile, or zBMI > + 2 SD). For studies conducted in specific countries or regions, classifications were based on locally authoritative standards. Racial subgroups were categorized as Black and other races. In addition, given their clinical relevance for exercise prescriptions, we conducted exploratory analyses including exercise type, frequency, intensity, intervention time, intervention period, and intervention type (comprehensive vs. single component). These analyses were intended to identify potential trends and generate hypotheses for future studies, rather than to test predefined hypotheses. To further explore potential sources of heterogeneity, univariable meta-regression analyses were performed. The included covariates were: exercise dose, training volume (weeks), exercise type, age, country type, and delivery setting. Meta-regression using exercise dose as a moderator was also conducted for other psychological outcomes to examine the relationship between exercise dose and mental health indicators. The exercise dose was calculated by multiplying the intervention frequency (sessions/week), intensity (measured in metabolic equivalents [METs]), and duration (minutes/session), and was expressed as MET-min/week. MET values were estimated using the Youth Compendium of Physical Activities, which provided a comprehensive list of 196 common physical activities for children and adolescents across different age groups [12]. Country type was classified according to the World Bank’s income group system. High-income countries were categorized as developed countries, while low-income, lower-middle-income, and upper-middle-income countries were grouped as developing countries [35]. Regarding the delivery setting, this study categorized exercise interventions into school-based physical activity and non-school-based physical activity. Sensitivity analysis was conducted by iteratively excluding individual studies to evaluate their impact on the overall findings [36, 37]. In addition, Baujat plots were generated to identify studies contributing most to heterogeneity and influencing the pooled effect by visualizing each study’s contribution to residual heterogeneity (x-axis) and its influence on the overall pooled estimate (y-axis). The certainty of evidence for each outcome was evaluated using the Grading of Recommendations Assessment, Development and Evaluation (GRADE), considering risk of bias, inconsistency, indirectness, imprecision, and publication bias. A Summary of Findings table was generated accordingly. All analyses were performed using Review Manager 5.3 [38], Stata 15.1, R v4.41, and GraphPad Prism9.5.

## Results

### Literature selection

**Fig. 1:**
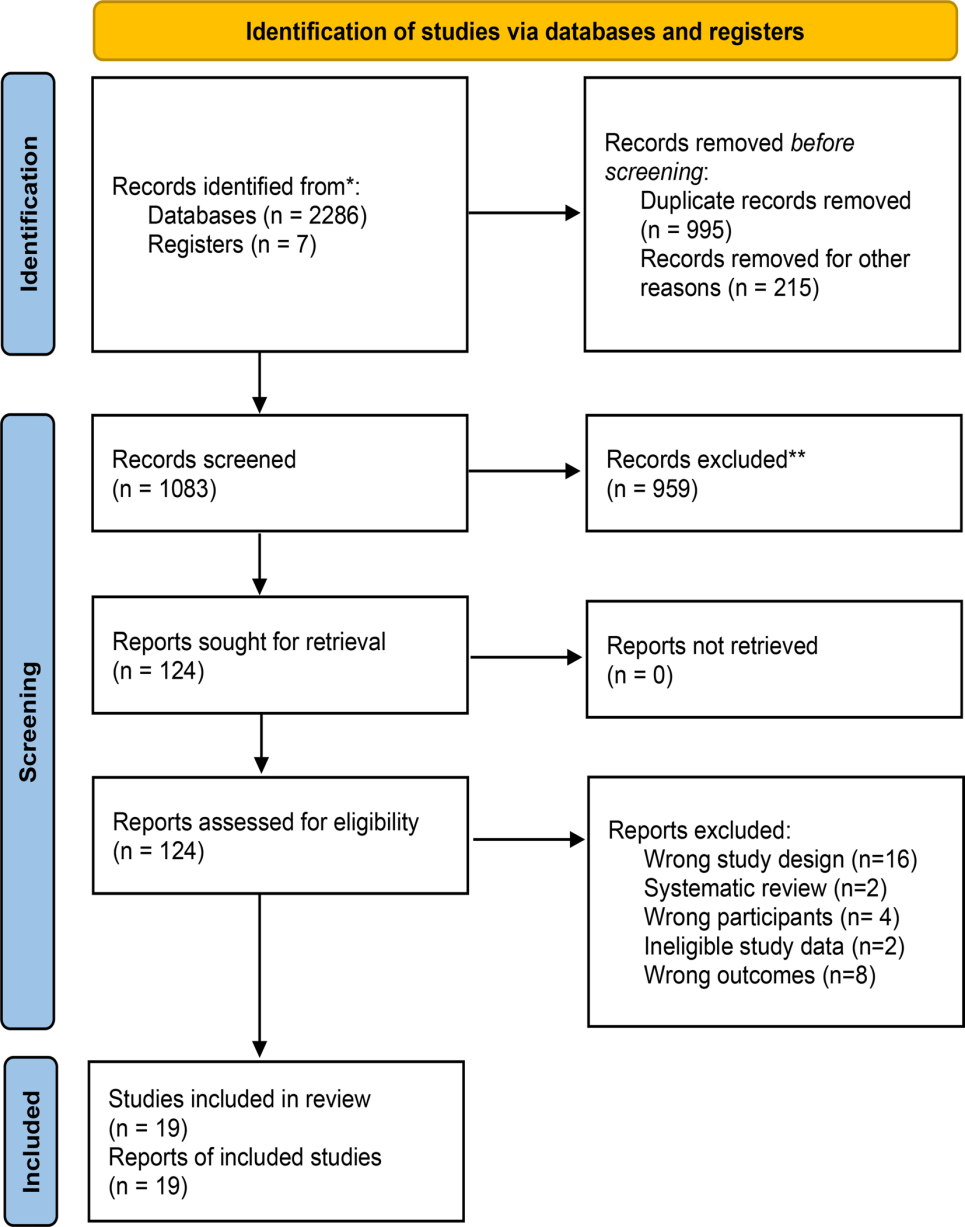
Preferred reporting items for systematic reviews and meta-analyses (PRISMA) 2020 flow diagram depicting the study selection process. Of the reports assessed for eligibility, 19 studies were included in the quantitative synthesis. Other reports were excluded owing to non-quantitative outcomes or insufficient data for effect size estimation

The systematic search identified 2,286 potentially relevant records through database retrieval, supplemented by 7 additional studies identified via manual searching. Following duplicate removal (*n* = 1,083) and rigorous multi-stage screening, 19 studies [39–57] meeting predefined inclusion criteria were included in the final analysis, encompassing a total of 1,795 participants. During the literature screening process, the inter-rater reliability was almost perfect (Cohen’s kappa = 0.85 at the title/abstract screening stage; Cohen’s kappa = 0.90 at the full-text screening stage). The complete literature selection process is detailed in Fig. 1 (PRISMA 2020 Flow Diagram). Appendix File 4 excluded studies with specific rationale for each exclusion decision, consistent with PRISMA reporting guidelines.

### Characteristics of the included studies

A total of 19 RCTs, involving 1,795 participants, were included in this study. The publication dates of the studies spanned from 2004 to 2023, with the majority of interventions conducted between 2010 and 2022, reflecting the contemporary rise in obesity and mental health concerns. The distribution of study timelines exhibited no notable geographical bias. 4 studies enrolled both overweight and obese participants (*n* = 228) [47, 50, 51, 53], 11 studies were limited to obese individuals (*n* = 996) [39, 40, 42–44, 49–55, 57], and 4 studies included only overweight participants (*n* = 571) [42, 48, 54, 55]. Of these, 8 studies involved adolescent populations (*n* = 835) [40, 42, 43, 46, 50–53], whereas 11 studies focused on children (*n* = 960) [39, 41, 44, 45, 47–49, 54–57]. 15 studies included mixed-gender participants (*n* = 1,456) [39–41, 43, 45–49, 51, 52, 54–57], 3 studies were exclusively female (*n* = 290) [42, 44, 53], and 1 study was exclusively male (*n* = 49) [50]. 2 studies exclusively targeted Black populations (*n* = 170) [51, 55], while 1 study divided participants into separate experimental groups for Black (*n* = 122) and White individuals (*n* = 85) [48]. The remaining 16 studies did not specify racial or ethnic groups (*n* = 1,418) [39–47, 49, 50, 52–54, 56, 57]. In terms of exercise types, 14 studies focused on aerobic exercise (*n* = 707) [39–42, 45, 46, 48, 49, 51, 52, 54–57], 1 study involved resistance training (*n* = 26) [50], and 3 studies combined exercise (*n* = 109) [44, 47, 53]. Additionally, 1 study included three experimental groups: aerobic exercise (*n* = 75), resistance training (*n* = 78), and combined exercise [43]. Of the 19 included studies, 7 featured intervention periods of ≤ 12 weeks (*n* = 202) [40, 41, 44, 45, 52, 53, 57], whereas 12 studies had intervention periods exceeding 12 weeks (*n* = 868) [39, 42, 43, 46–51, 54–56]. In terms of exercise session duration, 9 studies involved sessions lasting ≤ 45 min (*n* = 687) [39–43, 48, 51, 52, 55], and 10 studies involved sessions lasting > 45 min (*n* = 383) [44–47, 49, 50, 53, 54, 56, 57]. Regarding exercise frequency, 12 studies implemented ≤ 5 sessions per week (*n* = 735) [40, 42–44, 46, 47, 49, 50, 52–54, 56], and 7 studies implemented > 5 sessions per week (*n* = 335) [39, 41, 45, 48, 51, 55, 57]. 1 study categorized participants into distinct experimental groups based on intensity: moderate-to-low intensity (*n* = 69) and high intensity (*n* = 70) [48]. The remaining 15 studies applied moderate-to-low-intensity interventions (*n* = 647) [39–42, 44–46, 49–53, 55–57], and 3 studies applied high-intensity interventions (*n* = 284) [43, 47, 54]. Only 5 studies (26%) assessed intervention adherence [43, 45, 50–52]. Of these, two studies reported 100% adherence [45, 52], one reported 72% [50], one reported 62% [51], and one study reported adherence separately for different groups, with rates of 56%, 62%, and 64% across the three groups [43]. Ten studies described safety measures in detail [41–45, 47, 51, 53, 55, 56], whereas the remaining nine did not provide such information [39, 40, 46, 48–50, 52, 54, 57]. Additionally, 15 studies reported participant dropouts due to personal reasons [39–42, 44, 46–48, 50–53, 55–57], involving a total of 206 individuals. No adverse events related to the intervention itself were reported during the trials. Additional study characteristics are presented in Appendix File 5–7.

### Quality assessment

**Fig. 2:**
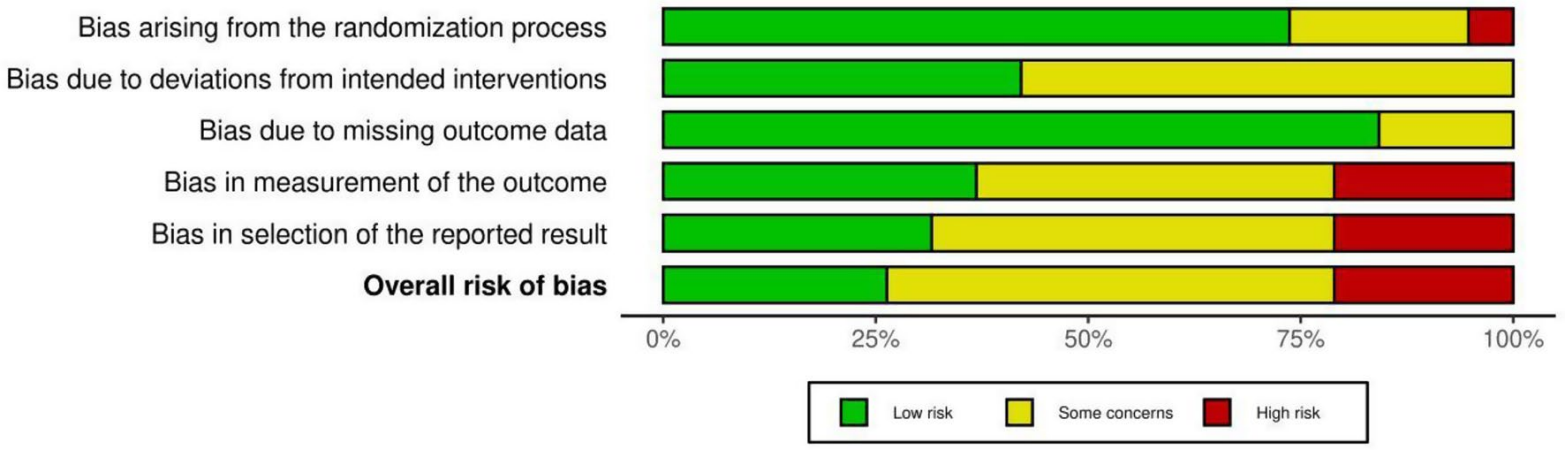
Risk of bias graph

**Fig. 3:**
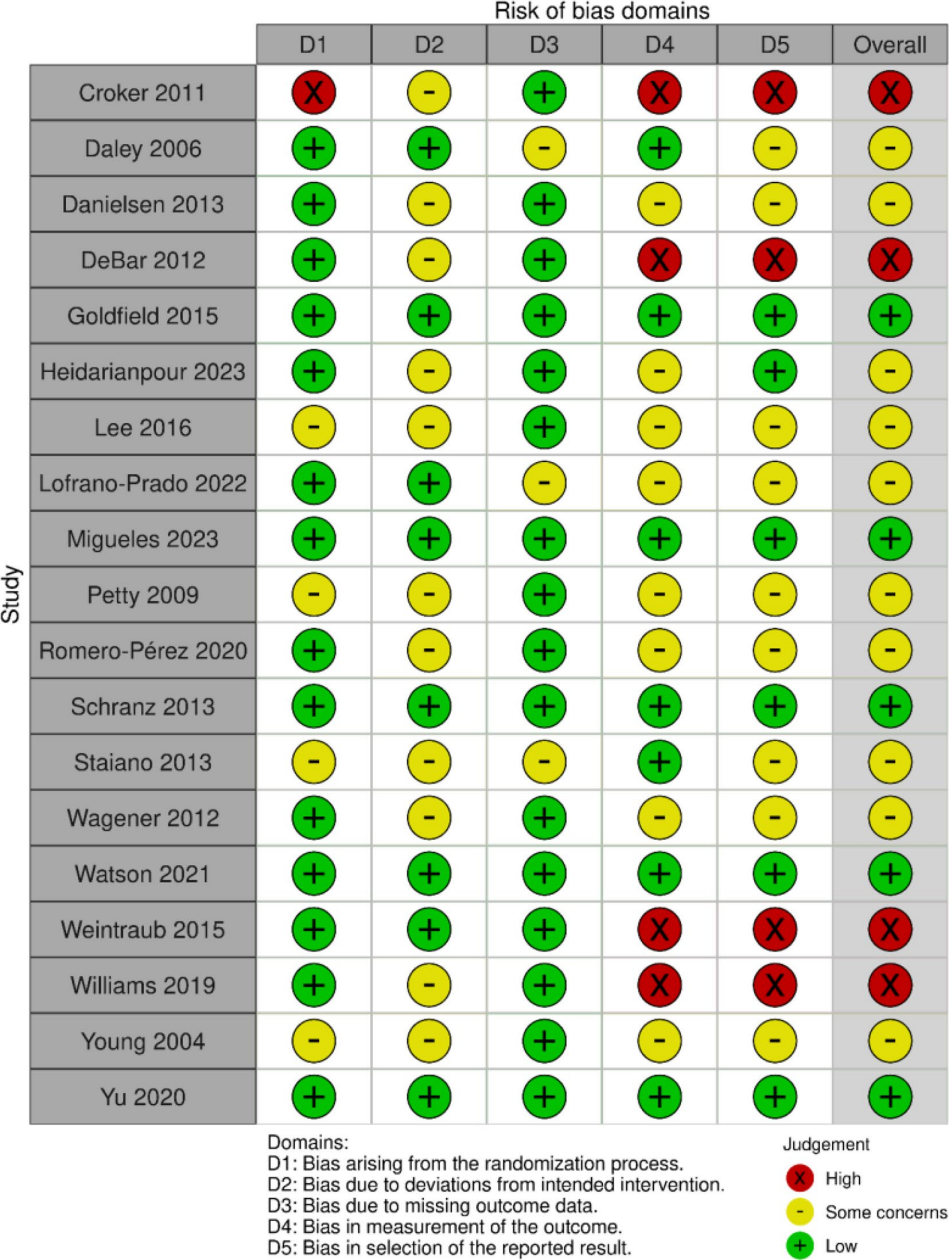
Risk of bias summary

In the assessment of the overall risk of bias for the 19 randomized controlled trials included, five studies (26.3%) were judged to have a low risk of bias, ten studies (52.6%) were assessed as having some concerns, and four studies (21.1%) were rated as having a high risk of bias. By domain, five studies (26.3%) showed bias arising from the randomization process; eleven studies (57.9%) had bias due to deviations from intended interventions; three studies (15.8%) had bias due to missing outcome data; twelve studies (63.4%) had bias in the measurement of outcomes; and thirteen studies (68.4%) showed bias in the selection of the reported result. The quality assessment results are illustrated in Figs. 2 and 3, with detailed evaluations for each study available in Appendix File 8.

### Meta-analysis results

Compared with the control group, the PA group showed a statistically significant reduction in anxiety (SMD(Hedges’ g) = -0.98, 95% CI -1.90 to -0.05, 95% PI -4.02 to 2.06, *P* = 0.04, *I²* = 95%). Although the point estimate suggested a large effect, the certainty of evidence was low, reflecting extreme heterogeneity across studies. The reduction in depression was also statistically significant (Hedges’ g = -0.15, 95% CI -0.25 to -0.05, 95% PI -0.25 to -0.05, *P* = 0.005, *I²* = 0%), representing a small effect of modest clinical importance. Regarding positive psychological outcomes, statistically significant improvements were observed in self-esteem (Hedges’ g = 0.19, 95% CI 0.03 to 0.35, 95% PI 0.03 to 0.35, *P* = 0.02, *I²* = 0%), representing a small effect, and in self-worth (Hedges’ g = 0.34, 95% CI 0.19 to 0.49, 95% PI 0.16 to 0.52, *P* = 0.0002, *I²* = 2%), indicating a small-to-moderate effect, although the certainty of evidence was low. Detailed meta-analysis results are presented in Appendix File 9–10. With the exception of anxiety (*I*² = 95%), no significant statistical heterogeneity was detected for the other outcomes. Anxiety (Hedges’ g = -0.98, 95% CI -1.90 to -0.05), heterogeneity was extreme (τ² = 1.69; *I²* = 94.89%). The 95% prediction interval was wide and crossed the null (-4.02 to 2.06). Sensitivity analyses using a leave-one-out approach showed that the pooled anxiety effect was not fully robust for this outcome; the 95% confidence interval crossed the null in some iterations. In addition, the Baujat plot suggested that Heidarianpour et al. 2023 contributed relatively more to the observed heterogeneity and had a greater influence on the pooled anxiety effect than other studies. Detailed leave-one-out and Baujat plot results for anxiety are provided in Appendix File 11.

**Fig. 4:**
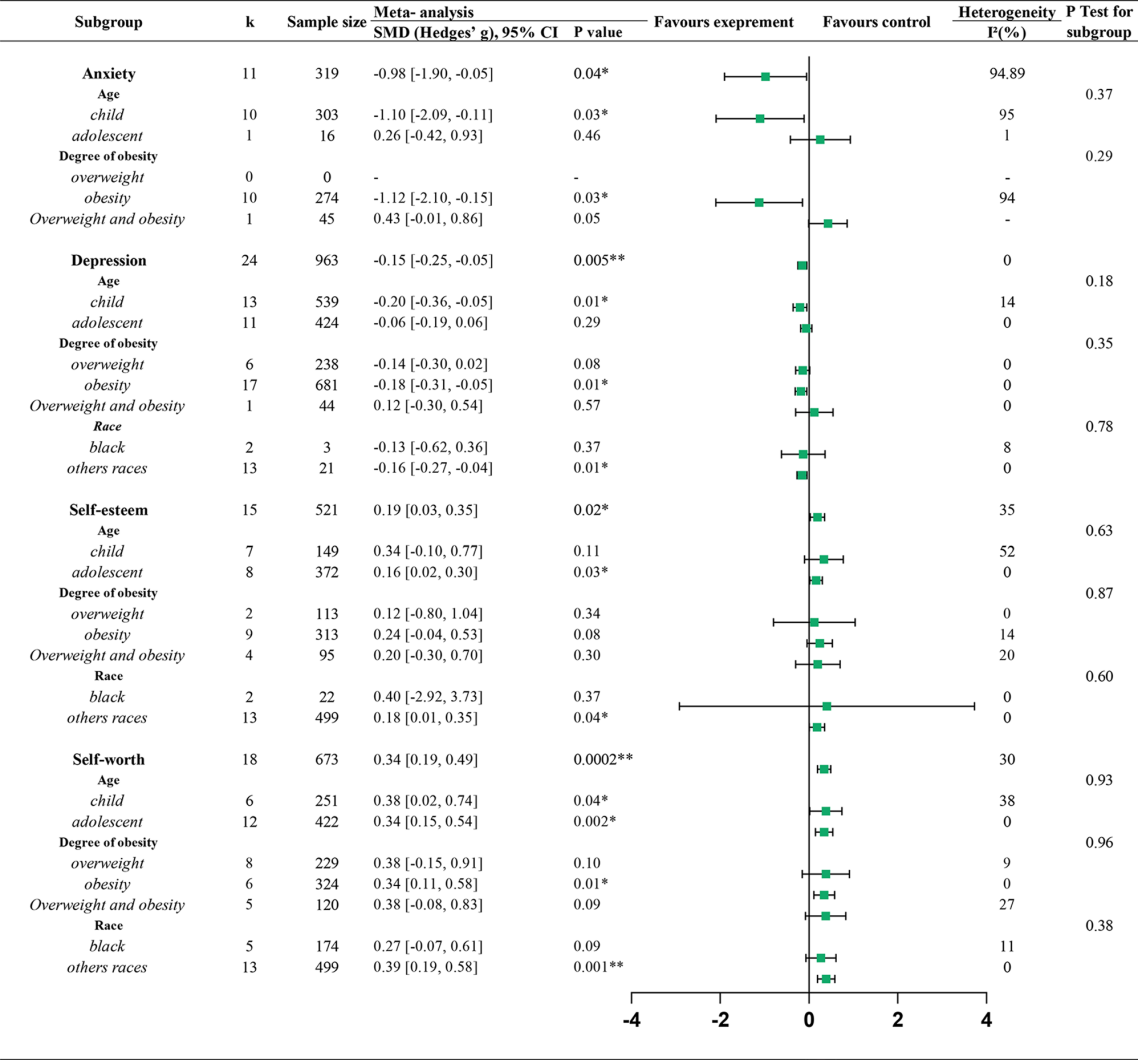
Potential moderators of the effects of physical activity on mental health in overweight/obese children and adolescents

To further explore factors potentially affecting the impact of PA interventions on mental health, we conducted subgroup analyses by age, obesity subtype, and racial categories, as shown in Fig. 4. Subgroup analyses by age revealed that PA led to more statistically significant improvements in anxiety (Hedges’ g = -1.10, -2.09 to -0.11; *P* = 0.03) and depression (Hedges’ g = -0.20, -0.36 to -0.05; *P* = 0.01) for children. For self-esteem, adolescents showed a statistically significant improvement (Hedges’ g = 0.16, 0.02 to 0.30; *P* = 0.03), while the effect in children was borderline (Hedges’ g = 0.34, -0.10 to 0.77; *P* = 0.11). Self-worth improved in both children (Hedges’ g = 0.38, 0.02 to 0.74; *P* = 0.04) and adolescents (Hedges’ g = 0.34, 0.15 to 0.54; *P* = 0.002). By obesity subtype indicated that PA resulted in greater improvements in depression (Hedges’ g = -0.18, -0.31 to -0.05; *P* = 0.01) and self-esteem (Hedges’ g = 0.24, -0.04 to 0.53; *P* = 0.08) among **obesity** subgroup than in their overweight subgroup. Subgroup analyses based on race revealed that, in contrast to other racial groups, Black participants did not show statistically significant improvements for depression (Hedges’ g = -0.13, -0.62 to 0.36; *P* = 0.37), self-esteem (Hedges’ g = 0.40, -2.92 to 3.73; *P* = 0.37), and self-worth (Hedges’ g = 0.27, -0.07 to 0.61; *P* = 0.09). Detailed subgroup analysis results are presented in Appendix File 12–16. Exploratory subgroup analyses by intervention type (comprehensive vs. single-component) showed the following: anxiety decreased statistically significantly with single-component interventions (Hedges’ g = -1.29, -2.32 to -0.25; *P* = 0.02). Depressive symptoms improved with comprehensive interventions (Hedges’ g = -0.20, -0.39 to -0.01; *P* = 0.04) and were borderline with single-component interventions (Hedges’ g = -0.12, -0.25 to 0.01; *P* = 0.06). Self-esteem increased under both approaches (comprehensive: Hedges’ g = 0.36, -0.14 to 0.85; *P* = 0.12; single-component: Hedges’ g = 0.15, -0.01 to 0.32; *P* = 0.06). Self-worth also improved in both subgroups (comprehensive: Hedges’ g = 0.63, 0.22 to 1.03; *P* = 0.01; single-component: Hedges’ g = 0.27, 0.12 to 0.41, *P* = 0.002). Detailed exploratory subgroup results are provided in Appendix File 17.

We performed a series of univariable random-effects meta-regression analyses to examine the influence of covariates on anxiety outcomes (see Additional Files 18–19). Country type (*β* = -1.76, *p* = 0.02) was a statistically significant covariate for anxiety symptoms. This result indicated that studies conducted in developing countries were associated with statistically significantly lower effect sizes compared to those in developed countries. Covariates such as exercise dose (*β* = 0.00, *p* = 0.74), training volume (*β* = 0.04, *p* = 0.66), mean age (*β* = 0.41, *p* = 0.43), type of intervention (aerobic vs. mixed: *β* = 1.18, *p* = 0.28), and single intervention vs. comprehensive intervention (*β* = -0.59, *p* = 0.59) had no statistically significant effect on anxiety symptoms.

Additionally, we examined the association between exercise dose (MET-min/week) and other psychological outcomes (depression, self-esteem, and self-worth). None of these associations reached statistical significance (depression: *β* = 0.00, *p* = 0.17; self-esteem: *β* = -0.15, *p* = 0.12; self-worth: *β* = 0.00, *p* = 0.97).

### Sensitivity analysis and publication bias assessment

Sensitivity analyses were conducted to assess the robustness of our findings. First, following the Cochrane Handbook recommendations, we set the correlation coefficient (Corr) to 0.5 for the primary analysis and performed an in-depth sensitivity analysis by varying the Corr value across a plausible range (0.3, 0.5, 0.7). The results demonstrated that the direction and statistical significance of the effects for all primary outcomes remained consistent across this range (see Appendix File 20), indicating robustness. Considering that adherence might influence the intervention effect and was reported in only five studies, we excluded these studies in a sensitivity analysis. The results remained consistent, indicating good robustness of the findings. Two studies were excluded from the meta-analysis due to insufficient data—one did not report standard deviations [58], and the other presented only pre–post mean changes without a control comparison [59]. Both studies showed within-group improvements consistent with the overall direction of our findings. Furthermore, we conducted an additional sensitivity analysis including only studies in which PA was the sole between-group difference, excluding trials with any additional intervention differences in diet, nutrition, psychological support, or other lifestyle factors. The results demonstrated that the pooled effect sizes, effect directions, and statistical significance were consistent with the primary analyses, indicating good robustness of the overall conclusions. Detailed information on the relevant trials is provided in the Appendix Files 21.

Publication bias and small-study effects were assessed using funnel plot inspection, Egger’s linear regression test, and the trim-and-fill method. Formal funnel-asymmetry testing (Egger’s test) was restricted to outcomes with at least 10 studies (k ≥ 10); therefore, Egger’s tests were conducted for anxiety (k = 11), depression (k = 23). self-esteem (k = 15) and self-worth (k = 18). All results from funnel asymmetry testing and trim-and-fill were interpreted as exploratory rather than confirmatory.

Visual inspection of funnel plots suggested apparent asymmetry for anxiety and self-worth, a mild rightward tendency for self-esteem, and approximate symmetry for depression (Appendix File 22–23). In exploratory Egger’s regression analyses, evidence of small-study effects was observed for anxiety (intercept = -5.06, 95% CI -9.68 to -0.43, *p* = 0.04) and self-worth (intercept = 1.94, 95% CI 0.33 to 3.54, *p* = 0.02). For self-esteem, Egger’s test did not reach conventional significance (intercept = 1.28, 95% CI -0.14 to 2.70, *p* = 0.07). For depression, Egger’s test was not significant (intercept = -0.31, 95% CI -1.87 to 1.26, *p* = 0.69).

To explore the potential impact of small-study effects on pooled estimates, trim-and-fill analyses were performed. No studies were imputed for anxiety or depression (k₀ = 0). In contrast, four and two studies were imputed to the left side of the funnel plot for self-esteem and self-worth, respectively. After adjustment, the pooled effect size for self-esteem decreased from *g* = 0.19 (95% CI 0.07–0.31) to *g* = 0.12 (95% CI 0.01–0.24), and for self-worth from *g* = 0.35 (95% CI 0.22–0.48) to *g* = 0.32 (95% CI 0.19–0.45).

Taken together, these results provide exploratory indications that small-study effects may be present for anxiety and self-worth. Trim-and-fill adjustments led to only modest changes in pooled estimates for self-esteem and self-worth; however, these analyses do not confirm the absence of publication bias, and findings should be interpreted with appropriate caution. The funnel plots (including trim-and-fill–adjusted funnel plots) and the results of Egger’s tests and trim-and-fill analyses are presented in Appendix File 22–23.

## Discussion

This meta-analysis examined the impact of PA on anxiety and depressive symptoms among overweight and obese children and adolescents, including 19 RCTs involving 1,795 participants. Overall certainty of evidence ranged from low to moderate across outcomes. Detailed GRADE assessments are provided in Appendix File 24. The results indicated that PA may alleviate anxiety symptoms compared with the control group, although the certainty of evidence was low. Although a statistically significant reduction in depression was observed, its magnitude did not reach clinically meaningful levels. Meanwhile, PA was associated with small improvements in self-esteem and self-worth. These findings are consistent with those reported by Chen et al. [21]. Additionally, the effect sizes were moderated by age strata, obesity severity grades, and racial categories, potentially attributable to differences in baseline measures, supportive environments, psychological assessment criteria, and cultural backgrounds.

The high prevalence and diagnostic complexity of comorbid depression in overweight/obese children and adolescents inevitably lead to increased rates of underdiagnosis and undertreatment [8], a trend consistent with the high comorbidity rates observed in the included studies. The discrepancy between high detection rates and low treatment rates not only results in missed opportunities for early prevention and intervention but also directly exacerbates treatment challenges [60]. Further increasing the risk of adverse outcomes and doubling the disease burden [61]. This underscores the critical importance of early intervention. Improvements in anxiety (Hedges’ g = -0.98), depression (Hedges’ g = -0.15), self-esteem (Hedges’ g = 0.19), and self-worth (Hedges’ g = 0.34) among overweight/obese children and adolescents were associated with PA. Although our effect sizes were generally lower compared to those reported by Michael [18] and Francesco et al. [8], all findings demonstrated statistically significant outcomes (*P* < 0.05). The relatively small effect sizes observed in this study may be partly attributable to the baseline psychological assessment scores of the included population; however, this explanation still requires direct verification in future randomized controlled trials through pre-specified subgroup analyses or meta-regression. Empirical studies have demonstrated a negative association between overweight/obesity and anxiety/depression prevalence with physical inactivity [62]. The potential benefits of PA for anxiety and depression may be explained by multiple mechanisms, including physiological pathways such as the activation of dopamine signaling and reinforcement of reward systems [63], promotion of brain-derived neurotrophic factor (BDNF) secretion [64], normalization of the HPA axis, and improvements in inflammatory and metabolic profiles [65]. As these biomarkers were not measured in the included RCTs, the discussion of potential mechanisms in this paragraph is based on previous literature rather than derived from the results of this meta-analysis. In psychological pathways, PA may improve anxiety and depression through mechanisms such as enhancing self-efficacy, strengthening social support, improving emotional regulation, and promoting a positive body image [66]. However, while PA may play a mediating role in psychological symptom improvement, definitive causal mechanisms**-**particularly neurobiological pathways-cannot be established based on the current evidence. Future studies should integrate biomarker assessments with psychometric measures to test these physiological hypotheses and elucidate the specific mechanisms through which PA improves mental health in this population. Given the inherent individual variability in psychopathological trajectories and the multifactorial nature of mental health interventions, isolating PA’s independent effects on specific symptom clusters remains methodologically challenging.

We found that the effects of PA on overweight and obese children and adolescents appeared to exhibit an age-related pattern. PA appeared to be associated with greater improvements in anxiety and depressive symptoms among children. However, the study by Francesco et al. [8] reported that PA had a more pronounced effect on depressive symptoms in adolescents aged 13 and older, which contrasts with our findings. This discrepancy may be attributed to differences in the study populations: their study included children and adolescents with anxiety and depression, whereas our analysis focused on individuals with high BMI accompanied by anxiety and depressive symptoms. Accumulating meta-analytic evidence suggests that PA may confer relatively greater mental health benefits in children, potentially due to heightened neuroplasticity and metabolic adaptability, which may allow them to derive greater benefits from PA [67]. Conversely, adolescents experience hormonal fluctuations (e.g., changes in testosterone and estrogen levels) that may interfere with the physiological pathways by which PA modulates mood regulation [68]. Moreover, genetic factors exert a lesser influence on childhood depression compared to environmental factors [69]. These hypotheses are all based on previous studies and were not directly tested in this study. Simultaneously, adolescents encounter multifaceted psychological challenges, including academic stress, social conflicts, and body image concerns, which can contribute to the accumulation of anxiety and depressive symptoms. Consequently, the positive effects of PA may be attenuated by competing stressors, indicating that adolescent mental health issues may necessitate integrated and multifaceted intervention approaches. Psychosocial context is likewise important: parental and broader environmental support can facilitate participation and enhance psychological gains [45, 70], and such support may be more consistently available in childhood than adolescence [55]. Future interventions should be tailored to age-specific differences, incorporating strategies such as social interaction and personalized goals, while integrating multidimensional support, including mental health education and family involvement, to maximize effectiveness.

Subgroup analyses suggested that children and adolescents with obesity may derive greater psychological benefits from PA compared with those who were overweight. Initial psychometric profiling demonstrated marked baseline disparities: 56% of obese participants exhibited notable depressive symptoms, compared to only 7% of overweight participants. Following the intervention, a convergence in depression scores was observed, suggesting a potentially greater responsiveness to PA among youth with obesity, whereas associations between PA and depression appeared minimal among overweight participants [3, 71–73]. The dose-response relationship between BMI and depression is consistent with previous studies [3, 74]. Additionally, obese children and adolescents face weight-related stigma and discrimination, which often mediate through low self-esteem to further increase the risk of anxiety and depression [3, 75, 76]. Obesity has also been consistently associated with poorer health-related quality of life [3], which may partly explain why psychological improvements associated with PA appear more pronounced in this subgroup.

In the subgroup analysis focusing on Black children and adolescents with overweight or obesity, the pooled effect of PA on mental health outcomes did not reach statistical significance. However, the limited sample size (*n* = 292) may have reduced statistical power and should be taken into consideration. Multiple studies acknowledge race as an important moderating variable, yet findings regarding PA are inconsistent. For example, one study with a predominantly African American sample observed significant benefits of PA [48](Exercise effects on depressive symptoms and self-worth in overweight children: A randomized controlled trial), while another 8-month PA intervention for overweight children in a Black community found no significant advantage of exercise in improving self-worth [55](Exercise effects on quality of life, mood, and self-worth in overweight children: the SMART randomized controlled trial), which is also supported by another meta-analysis [77]. Existing literature suggests that potential sociocultural factors may influence the relationship between PA and mental health in this population. For instance, some studies indicate that the manifestation and correlates of depressive symptoms may differ across racial groups [78]. However, current psychometric evaluations still lack measurement invariance across races, which severely undermines the reliability of subgroup comparisons derived from them [79]. Furthermore, broader contextual factors, such as experiences of racial discrimination, have been identified as significant mediators for depressive symptoms in Black adolescents [80], which could potentially mask or moderate the effects of PA interventions, providing another explanation for the results. Therefore, the interpretation of the statistical results may not reflect the true underlying effect. Given the currently limited evidence, we are unable to draw firm conclusions regarding the potential effects of PA interventions on depression, self-esteem, or self-worth among Black children and adolescents, and these results should be interpreted with caution. Future research urgently requires larger, culturally diverse samples and assessment tools with established measurement invariance across racial groups to further validate the efficacy of PA in this population.

Gender disparities in depression often first manifest during adolescence [81]. However, due to the limited number of single-gender studies included in our analysis, we were unable to conduct subgroup analyses. Additionally, while the type of physical activity (e.g., aerobic exercise, resistance training, combined exercise) is a crucial factor to consider in its application, our subgroup analysis did not reveal significant differences based on exercise type (see Appendix File 14). Consequently, we cannot determine the influence of gender and exercise type on the effects of PA on anxiety and depression.

To explore potential sources of statistically significant heterogeneity (*I²* = 95%) in anxiety outcomes, we conducted a series of univariable meta-regression analyses. The results indicated that country type was the only statistically significant moderator (*β* = -1.76, *p* = 0.02). Studies conducted in developing countries reported significantly smaller effects of physical activity on anxiety compared with those in developed countries, which may be attributable to differences in study quality and implementation contexts. Although we used meta-regression to explore the dose-response relationship between PA and reductions in anxiety and depression scores (in Appendix File 16–17) and also tried to find an effective intervention cycle, intervention duration, intervention frequency, and intervention intensity through subgroup analysis (in Appendix File 14), the optimal exercise dose to improve anxiety and depression in overweight or obese children and adolescents has not been achieved. There are several possible reasons for this finding. First, the overall sample size of the included studies was relatively small. Second, the exercise dose was expressed in METs, and most studies involved mixed populations, making it difficult to account for inter-individual variability in exercise-induced thresholds. Third, most interventions involved low-to-moderate intensity exercise, which limited the ability of regression models to detect threshold or non-linear effects. Fourth, several studies lacked accurate information on participants’ daily exercise levels. Finally, heterogeneity in intervention characteristics-including family involvement, exercise type, dietary components, and reward settings-may have further contributed to the inconsistent findings. Given the extreme between-study heterogeneity and the wide prediction interval crossing the null, the pooled effect for anxiety should be interpreted cautiously. Although the average estimate suggests a reduction in anxiety, the prediction interval indicates that the true effect in a new setting could plausibly range from substantial benefit to little or no benefit, and potentially even harm. This pattern is consistent with the observed heterogeneity and suggests that the anxiety outcome is likely context-dependent rather than uniform across studies. Accordingly, conclusions were framed conservatively, with an emphasis on limited generalizability and context dependence.

## Strengths and limitations

This study demonstrates three significant strengths. First, its methodological rigor: the research rigorously followed PRISMA guidelines and was pre-registered on the PROSPERO platform, ensuring transparency and reproducibility. Second, its population specificity and high level of evidence: this study represents the first meta-analysis of RCTs assessing the impact of PA on anxiety and depression in overweight or obese children and adolescents, while also evaluating self-esteem and self-worth as social functional metrics. Third, subgroup analyses revealed different responses among populations - for example, greater anxiety reduction in obese children, lower PA responsiveness in Black participants, and a need for culturally adapted interventions. These results provide evidence to guide precision-based interventions and future policy development. Moreover, they will inspire further investigation into the underlying mechanisms, causes, and therapeutic approaches for anxiety and depression in overweight and obese children and adolescents.

In addition, this study has several limitations. First, there was evidence of publication bias and substantial heterogeneity. The funnel plots for some outcomes (e.g., anxiety and self-esteem) were asymmetric, and Egger’s tests suggested the presence of small-sample effects or publication bias. Although the trim-and-fill analysis indicated that the main conclusions remained robust, the results should still be interpreted with caution. Moreover, high heterogeneity was observed in the anxiety outcome (*I²* = 91%). Meta-regression analysis identified country type as a significant moderator, but other unmeasured factors may also have contributed to the residual heterogeneity. Second, issues related to psychometric instruments and cross-cultural measurement bias should be noted. The included studies used different scales to assess the same constructs, and variations in scale focus may have introduced measurement bias. Furthermore, the instruments may lack measurement equivalence across different cultural or ethnic groups, potentially compromising the validity of cross-cultural comparisons. Third, the age range of the included populations was wide. Although age subgroups were defined according to developmental theory, categorizing participants aged 5–19 years into only two broad subgroups (children and adolescents) may have masked within-group developmental differences. This is particularly relevant for the adolescent group (13–19 years), which included individuals at distinct stages of maturation, potentially contributing to heterogeneity. Fourth, some subgroup analyses were based on limited sample sizes. For instance, the Black subgroup included only three studies (*n* = 292), which may have reduced statistical power and calls for cautious interpretation of the results. Fifth, there was a lack of long-term follow-up data. Almost all included studies failed to report post-intervention follow-up outcomes, making it impossible to assess the sustainability of the psychological benefits of physical activity; therefore, the long-term efficacy remains unclear. Additionally, factors such as language bias due to the restriction to Chinese and English databases, the exploratory nature of certain subgroup analyses, potential participant overlap across studies, and the fact that most studies did not report intervention adherence may all have affected the precision and generalizability of the findings.

## Conclusion

The evidence from this meta-analysis suggests that PA may be associated with improvements in anxiety and depressive symptoms among children with overweight or obesity. Among adolescents, PA shows a more consistent association with improvements in self-esteem, while self-worth appears to improve in both children and adolescents. However, the strength and consistency of these associations vary across outcomes and populations. Overall, these findings suggest that PA may serve as a potential adjunctive strategy for mental health management in this population, rather than a substitute for existing psychological or pharmacological treatments. Future research should optimize psychological assessment tools with respect to age, obesity subtypes, and cultural backgrounds, standardize and report PA dose to examine dose–response relationships, and conduct long-term follow-up to assess psychological outcomes.

## Supplementary Information

Below is the link to the electronic supplementary material.

Supplementary Material 1

## Data Availability

Data supporting the findings of this study are included in this published article and its supplementary materials. The full per-study data extraction table and all analysis scripts are publicly available in the Zenodo repository (10.5281/zenodo.17929144).
