# Supplementary material for: Does physical activity really improve anxiety and depression in overweight or obese children and adolescents? A systematic review and meta-analysis
Source: BMC Psychiatry. 2026 Jan 16;26:139. doi: 10.1186/s12888-025-07761-9 (PMC12892821; doi:10.1186/s12888-025-07761-9)
Supplement: Supplementary file 1 — Supplementary Material 1 [file 12888_2025_7761_MOESM1_ESM.zip › Appendix/Additional file 10 Meta-analysis results for outcomes.docx]

**Additional file 10** Meta-analysis results for outcomes

| **Outcome** | **k** | **Hedges'g (SMD)** | **95% CI lower** | **95% CI upper** | **Tau²** | **Tau² estimator** | **Hartung–Knapp adjustment** | ***I²* (%)** | ***I²* 95% CI** | **95% Prediction Interval** |
| --- | --- | --- | --- | --- | --- | --- | --- | --- | --- | --- |
| Depression | 23 | -0.15 | -0.25 | -0.05 | 0 | REML | Yes (HKSJ/Knha) | 0.0 | 0.0–40.06% | (-0.25, -0.05) |
| Anxiety | 11 | -0.98 | -1.9 | -0.05 | 1.69 | REML | Yes (HKSJ/Knha) | 94.89 | 89.19–98.46% | (-4.02, 2.06) |
| Self-esteem | 15 | 0.19 | 0.03 | 0.35 | 0 | REML | Yes (HKSJ/Knha) | 0.0 | 0.0–72.79% | (0.03, 0.35) |
| Self-worth | 18 | 0.34 | 0.19 | 0.49 | 0 | REML | Yes (HKSJ/Knha) | 2.38 | 0.0–63.87% | (0.16, 0.52) |

*k* indicates the number of included studies. SMD (Hedges’ *g*) denotes the standardized mean difference. 95% CI represents the 95% confidence interval of the pooled effect. τ² is the between-study variance, estimated using restricted maximum likelihood (REML). *I*² indicates the proportion of total variability due to between-study heterogeneity, with corresponding 95% CI. The 95% prediction interval reflects the range in which the true effect of a future study is expected to lie. Confidence intervals were calculated using the Hartung–Knapp–Sidik–Jonkman adjustment.
