# Supplementary material for: Does physical activity really improve anxiety and depression in overweight or obese children and adolescents? A systematic review and meta-analysis
Source: BMC Psychiatry. 2026 Jan 16;26:139. doi: 10.1186/s12888-025-07761-9 (PMC12892821; doi:10.1186/s12888-025-07761-9)
Supplement: Supplementary file 1 — Supplementary Material 1 [file 12888_2025_7761_MOESM1_ESM.zip › Appendix/Additional file 11 Sensitivity analysis forest plots for anxiety and Baujat plot results.docx]

Additional file 11 Sensitivity analysis forest plots for anxiety and Baujat plot results

| Anxiety | SMD (Hedges’ g) | 95%CI | *P* |
| --- | --- | --- | --- |
| Omitting Heidarianpour et al. 2023(1) | -0.72 | [-1.54, 0.09] | 0.08 |
| Omitting Heidarianpour et al. 2023(2) | -0.90 | [-1.93, 0.12] | 0.08 |
| Omitting Heidarianpour et al. 2023(3) | -0.92 | [-1.94, 0.11] | 0.07 |
| Omitting Heidarianpour et al. 2023(4) | -1.11 | [-2.09, -0.12] | 0.03* |
| Omitting Heidarianpour et al. 2023(5) | -1.05 | [-2.08, -0.03] | 0.05 |
| Omitting Heidarianpour et al. 2023(6) | -0.86 | [-1.85, 0.13] | 0.08 |
| Omitting Heidarianpour et al. 2023(7) | -0.82 | [-1.78, 0.14] | 0.08 |
| Omitting Migueles et al. 2023 | -1.12 | [-2.10, -0.15] | 0.03* |
| Omitting Romero-Pérez et al. 2020 | -1.11 | [-2.10, -0.11] | 0.03* |
| Omitting Wagener et al. 2012 | -1.10 | [-2.09, -0.11] | 0.03* |
| Omitting Yu et al. 2020 | -1.03 | [-2.07, 0.01] | 0.05 |

Effect sizes are SMD (Hedges’ g). CI, confidence interval. (1)-(7) indicate multiple outcome subscales from the same study. *P < 0.05 indicates statistical significance.


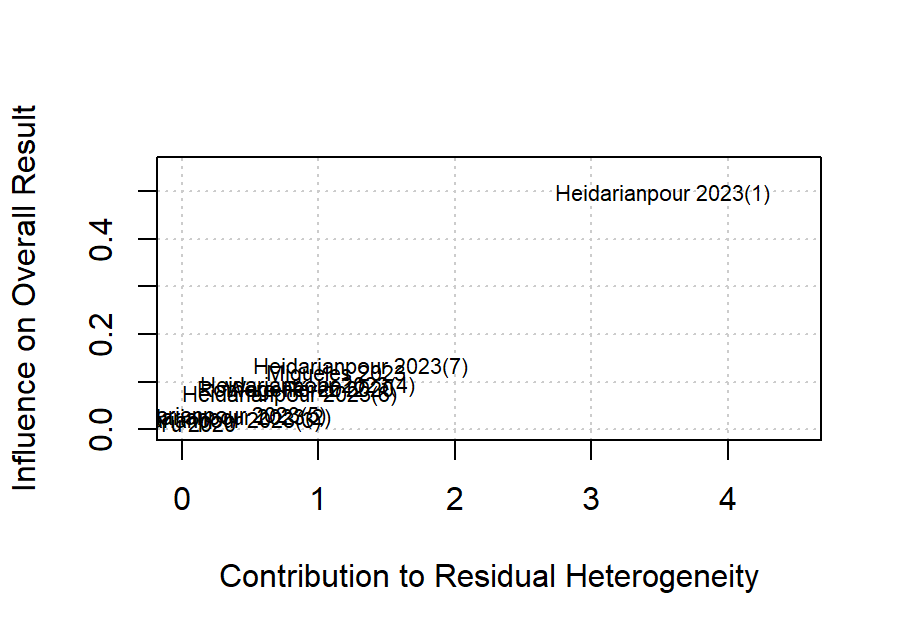


Each point represents a single study. The horizontal axis indicates the contribution of each study to the overall heterogeneity, while the vertical axis represents its influence on the pooled standardized mean difference (SMD).
