# Supplementary material for: Does physical activity really improve anxiety and depression in overweight or obese children and adolescents? A systematic review and meta-analysis
Source: BMC Psychiatry. 2026 Jan 16;26:139. doi: 10.1186/s12888-025-07761-9 (PMC12892821; doi:10.1186/s12888-025-07761-9)
Supplement: Supplementary file 1 — Supplementary Material 1 [file 12888_2025_7761_MOESM1_ESM.zip › Appendix/Additional file 12 Statistical table of predefined subgroup analysis results.docx]

**Additional file 12** Statistical table of predefined subgroup analysis results

| Outcomes | Subgroup | | k | SMD (Hedges’ g), 95% CI | *P* value | *I²*(%) | *P* Test for subgroup differences |
| --- | --- | --- | --- | --- | --- | --- | --- |
| Anxiety | Age | children（5-12-year-old） | 10 | -1.10 [-2.09, -0.11] | 0.03* | 95 | 0.37 |
|  |  | adolescent（12-19-year-old） | 1 | 0.26 [-0.42, 0.93] | 0.46 | - |  |
|  | Degree of obesity | overweight | 0 | - | - | - | 0.29 |
|  |  | obesity | 10 | -1.12 [-2.10, -0.15] | 0.03* | 94 |  |
|  |  | overweight and obesity | 1 | 0.43 [-0.01, 0.86] | 0.05 | - |  |
|  | Race | black | - | - | - | - | - |
|  |  | others races | - | - | - | - |  |
| Depression | Age | children（5-12-year-old） | 13 | -0.20 [-0.36, -0.05] | 0.01* | 14 | 0.18 |
|  |  | adolescent（12-19-year-old） | 11 | -0.06 [-0.19, 0.06] | 0.29 | 0 |  |
|  | Degree of obesity | overweight | 6 | -0.14 [-0.30, 0.02] | 0.08 | 0 | 0.35 |
|  |  | obesity | 17 | -0.18 [-0.31, -0.05] | 0.01* | 0 |  |
|  |  | overweight and obesity | 1 | 0.12 [-0.30, 0.54] | 0.57 | - |  |
|  | Race | black | 3 | -0.13 [-0.62, 0.36] | 0.37 | 8 | 0.78 |
|  |  | others races | 21 | -0.16 [-0.27, -0.04] | 0.01* | 0 |  |
| Self-esteem | Age | children（5-12-year-old） | 7 | 0.34 [-0.10, 0.77] | 0.11 | 52 | 0.63 |
|  |  | adolescent（12-19-year-old） | 8 | 0.16 [0.02, 0.30] | 0.03* | 0 |  |
|  | Degree of obesity | overweight | 2 | 0.12 [-0.80, 1.04] | 0.34 | 0 | 0.87 |
|  |  | obesity | 9 | 0.24 [-0.04, 0.53] | 0.08 | 14 |  |
|  |  | overweight and obesity | 4 | 0.20 [-0.30, 0.70] | 0.30 | 20 |  |
|  | Race | black | 2 | 0.40 [-2.92, 3.73] | 0.37 | 0 | 0.60 |
|  |  | others races | 13 | 0.18 [0.01, 0.35] | 0.04* | 0 |  |
| self-worth | Age | children（5-12-year-old） | 6 | 0.38 [0.02, 0.74] | 0.04* | 38 | 0.93 |
|  |  | adolescent（12-19-year-old） | 12 | 0.34 [0.15, 0.54] | 0.002* | 0 |  |
|  | Degree of obesity | overweight | 4 | 0.38 [-0.15, 0.91] | 0.10 | 9 | 0.96 |
|  |  | obesity | 8 | 0.34 [0.11, 0.58] | 0.01* | 0 |  |
|  |  | overweight and obesity | 6 | 0.38 [-0.08, 0.83] | 0.09 | 27 |  |
|  | Race | black | 5 | 0.27 [-0.07, 0.61] | 0.09 | 11 | 0.38 |
|  |  | others races | 13 | 0.39 [0.19, 0.58] | 0.001** | 0 |  |

k indicates the number of included studies. SMD (Hedges’ g) denotes the standardized mean difference. 95% CI represents the 95% confidence interval of the pooled effect. *I²* indicates the proportion of total variability due to between-study heterogeneity, with corresponding 95% CI. *P* < 0.05 was considered statistically significant, and *P* < 0.01 indicates high statistical significance. **P*＜0.05，***P*＜0.01
