# Supplementary material for: Does physical activity really improve anxiety and depression in overweight or obese children and adolescents? A systematic review and meta-analysis
Source: BMC Psychiatry. 2026 Jan 16;26:139. doi: 10.1186/s12888-025-07761-9 (PMC12892821; doi:10.1186/s12888-025-07761-9)
Supplement: Supplementary file 1 — Supplementary Material 1 [file 12888_2025_7761_MOESM1_ESM.zip › Appendix/Additional file 17 Statistical table of exploratory subgroup analysis results.docx]

**Additional file 1****7** Statistical table of exploratory subgroup analysis results

| **Outcomes** | **Subgroup** | | **k** | **Hedges’ g (SMD), 95% CI** | ***P* value** | ***I²* (%)** | ***P* Test for subgroup differences** |
| --- | --- | --- | --- | --- | --- | --- | --- |
| Anxiety | Exercise frequency | ≤5 times a week | - | - | - | - | ***-*** |
|  |  | ＞5 times a week | - | - | - | - |  |
|  | Intervention period | ≤12weeks | 2 | -1.39 [-2.58, -0.21] | 0.03* | 91 | 0.12 |
|  |  | ＞12weeks | 3 | 0.03 [-1.24, 1.30] | 0.94 | 86 |  |
|  | Intervention time | ≤45min | 1 | 0.26 [-0.42, 0.93] | 0.46 | - | 0.37 |
|  |  | ＞45min | 4 | -1.10 [-2.09, -0.11] | 0.03* | 95 |  |
|  | Intervention intensity | low and medium | - | - | - | - | - |
|  |  | high | - | - | - | - |  |
|  | Exercise type | aerobic exercise | 3 | -0.05 [-1.20, 1.09] | 0.86 | 79 | 0.15 |
|  |  | aerobic and resistance exercise | 2 | -1.37 [-2.58, -0.15] | 0.03* | 93 |  |
|  | Intervention type | comprehensive | 2 | 0.32 [-0.93, 1.56] | 0.19 | 0 | 0.13 |
|  |  | single-component | 3 | -1.29 [-2.32, -0.25] | 0.02* | 93 |  |
|  | Type of national development | developed country | 3 | 0.31 [0.02, 0.59] | 0.04* | 0 | 0.04* |
|  |  | developing country | 2 | -1.48 [-2.57, -0.39] | 0.01* | 92 |  |
| Depression | Exercise frequency | ≤5 times a week | 10 | -0.15 [-0.27, -0.04] | 0.01* | 0 | 0.87 |
|  |  | ＞5 times a week | 4 | -0.15 [-0.39, 0.09] | 0.18 | 10 |  |
|  | Intervention period | ≤12weeks | 5 | -0.19 [-0.44, 0.05] | 0.11 | 0 | 0.59 |
|  |  | ＞12weeks | 9 | -0.13 [-0.24, -0.03] | 0.02* | 0 |  |
|  | Intervention time | ≤45min | 6 | -0.13 [-0.24, -0.01] | 0.03* | 0 | 0.59 |
|  |  | ＞45min | 8 | -0.17 [-0.41, 0.06] | 0.12 | 22 |  |
|  | Intervention intensity | low and medium | 10 | -0.16 [-0.31, -0.01] | 0.04* | 9 | 0.76 |
|  |  | high | 4 | -0.13 [-0.26, 0.00] | 0.06 | 0 |  |
|  | Exercise type | aerobic exercise | 12 | -0.16 [-0.26, -0.05] | 0.004** | 0 | 0.64 |
|  |  | resistance exercise | 1 | -0.28 [-0.73, 0.17] | 0.22 | - |  |
|  |  | aerobic and resistance exercise | 3 | -0.14 [-1.34, 1.05] | 0.65 | 64 |  |
|  | Intervention type | comprehensive | 6 | -0.20 [-0.39, -0.01] | 0.04* | 1 | 0.41 |
|  |  | single-component | 9 | -0.12 [-0.25, 0.01] | 0.06 | 0 |  |
| Self-esteem | Exercise frequency | ≤5 times a week | 7 | 0.24 [0.04, 0.43] | 0.02* | 0 | 0.21 |
|  |  | ＞5 times a week | 2 | 0.01 [-0.36, 0.38] | 0.94 | 0 |  |
|  | Intervention period | ≤12weeks | 2 | 0.48 [-0.19, 1.15] | 0.12 | 50 | 0.09 |
|  |  | ＞12weeks | 7 | 0.13 [0.01, 0.25] | 0.04* | 0 |  |
|  | Intervention time | ≤45min | 5 | 0.23 [0.02, 0.43] | 0.03* | 0 | 0.42 |
|  |  | ＞45min | 4 | 0.08 [-0.33, 0.49] | 0.56 | 0 |  |
|  | Intervention intensity | low and medium | 6 | 0.30 [0.01, 0.59] | 0.04* | 34 | 0.44 |
|  |  | high | 3 | 0.12 [-0.07, 0.31] | 0.16 | 0 |  |
|  | Exercise type | aerobic exercise | 6 | 0.24 [-0.05, 0.53] | 0.09 | 26 | 0.54 |
|  |  | resistance exercise | 2 | 0.33 [-0.74, 1.40] | 0.16 | 0 |  |
|  |  | aerobic and resistance exercise | 3 | 0.05 [-0.29, 0.40] | 0.65 | 0 |  |
|  | Intervention type | comprehensive | 3 | 0.36 [-0.14, 0.85] | 0.12 | 59 | 0.55 |
|  |  | single-component | 6 | 0.15 [-0.01, 0.32] | 0.06 | 0 |  |
| self-worth | Exercise frequency | ≤5 times a week | 5 | 0.34 [0.15, 0.54] | 0.002** | 0 | 0.93 |
|  |  | ＞5 times a week | 2 | 0.38 [0.02, 0.74] | 0.04* | 38 |  |
|  | Intervention period | ≤12weeks | 3 | 0.53 [0.19, 0.86] | 0.01* | 10 | 0.11 |
|  |  | ＞12weeks | 4 | 0.26 [0.10, 0.43] | 0.01* | 0 |  |
|  | Intervention time | ≤45min | 5 | 0.30 [0.14, 0.46] | 0.001** | 0 | 0.25 |
|  |  | ＞45min | 2 | 0.56 [-0.01, 1.14] | 0.05 | 0 |  |
|  | Intervention intensity | low and medium | 5 | 0.43 [0.17, 0.69] | 0.004** | 26 | 0.47 |
|  |  | high | 2 | 0.29 [0.08, 0.50] | 0.01* | 0 |  |
|  | Exercise type | aerobic exercise | 5 | 0.34 [0.13, 0.55] | 0.004** | 16 | 0.97 |
|  |  | resistance exercise | 2 | 0.36 [-1.44, 2.17] | 0.24 | 0 |  |
|  |  | aerobic and resistance exercise | 2 | 0.40 [-0.20, 1.01] | 0.14 | 10 |  |
|  | Intervention type | comprehensive | 3 | 0.63 [0.22, 1.03] | 0.01* | 1 | 0.04* |
|  |  | single-component | 5 | 0.27 [0.12, 0.41] | 0.002** | 0 |  |

k indicates the number of included studies. SMD (Hedges’ g) denotes the standardized mean difference. 95% CI represents the 95% confidence interval of the pooled effect. *I²* indicates the proportion of total variability due to between-study heterogeneity, with corresponding 95% CI. *P* < 0.05 was considered statistically significant, and *P* < 0.01 indicates high statistical significance. **P*＜0.05，***P*＜0.01
