# Supplementary material for: Does physical activity really improve anxiety and depression in overweight or obese children and adolescents? A systematic review and meta-analysis
Source: BMC Psychiatry. 2026 Jan 16;26:139. doi: 10.1186/s12888-025-07761-9 (PMC12892821; doi:10.1186/s12888-025-07761-9)
Supplement: Supplementary file 1 — Supplementary Material 1 [file 12888_2025_7761_MOESM1_ESM.zip › Appendix/Additional file 18 Results of meta-regression analysis.docx]

Additional file 18 Results of meta-regression analysis

| **Outcome** | **Covariate (Ref)** | **β** | **SE** | **95% CI (lower, upper)** | **t** | ***p*** |
| --- | --- | --- | --- | --- | --- | --- |
| Anxiety | MET | 0.00024 | 0.00062 | (-0.00130, 0.00170) | 0.38 | 0.74 |
| Anxiety | Training volume | 0.037 | 0.073 | (-0.109, 0.184) | 0.51 | 0.66 |
| Anxiety | Mean age | 0.407 | 0.328 | (-0.053, 0.867) | 1.24 | 0.43 |
| Anxiety | Exercise type (Aerobic) | 1.18 | 0.89 | (-0.64, 3.00) | 1.33 | 0.28 |
| Anxiety | Country type (Developed) | -1.76 | 0.41 | (-3.19, -0.33) | -4.32 | 0.023* |
| Anxiety | Intervention type (Single-component) | -0.591 | 0.918 | (-2.935, 1.753) | -0.644 | 0.59 |
| Anxiety | Delivery setting (Non-school-based) | 0.59 | 1.2 | (-1.75, 2.94) | 0.49 | 0.62 |
| Depression | MET | 0.0001 | 0.00006 | (0.00000, 0.00020) | 1.58 | 0.17 |
| Self-esteem | MET | -0.152 | 0.079 | (-0.307, 0.003) | -1.93 | 0.123 |
| Self-worth | MET | -0.000003 | 0.000075 | (-0.000150, 0.000144) | -0.04 | 0.971 |
| MET, Metabolic Equivalent of Task; β (coefficient): Unstandardized meta-regression coefficient. For continuous moderators, β is the change in the pooled effect size per 1-unit increase; for categorical moderators, β is the difference from the reference group. Positive β means a larger effect as the moderator increases; negative β means a smaller effect, SE, Standard Error; CI, Confidence Interval; Ref, Reference Category;**P*＜0.05 | | | | | | |
