# Supplementary material for: Does physical activity really improve anxiety and depression in overweight or obese children and adolescents? A systematic review and meta-analysis
Source: BMC Psychiatry. 2026 Jan 16;26:139. doi: 10.1186/s12888-025-07761-9 (PMC12892821; doi:10.1186/s12888-025-07761-9)
Supplement: Supplementary file 1 — Supplementary Material 1 [file 12888_2025_7761_MOESM1_ESM.zip › Appendix/Additional file 19 Meta-regression information table.docx]

Additional file 19 Meta-regression information table

| Author | Duration (weeks) | Age | Activities | | | Category | METs | MET-min/week |
| --- | --- | --- | --- | --- | --- | --- | --- | --- |
|  |  |  | Type | Session length (min) | Frequency per week |  |  |  |
| Croker et al.[1] | 24 weeks | 10.8±1.6 | structured activity | 60 | 7 | Free play | 5.9 | 2478 |
| Daley et al.[2] | 8 weeks | 13.1 | Aerobic exercise (steping,cycling, rowing,dance mat and walking) | 30 | 3 | Jog self-paced | 7.9 | 711 |
| Danielsen et al.[3] | 12 weeks | 10.68±1.24 | aerobic exercise | 30 | 7 | Free play | 5.9 | 1239 |
| DeBar et al.[4] | 24 weeks | 14.12±1.48 | Yoga, Dance,Video Games | 30-60 | 5 | Aerobic dance/dance | 4.5 | 1350 |
| Goldfield et al.[5] | 22 weeks | 14-17 | aerobic exercise | 20-45 | 4 | Jog fast | 8.5 | 1530 |
|  |  |  | resistance training | 20-45 | 4 | Bench press | 1.9 | 342 |
|  |  |  | combine training | 20-45 | 4 | Free play | 6 | 1080 |
| Heidarianpour et al.[6] | 12 weeks | 8.51±0.44 | Combine training | 60 | 3 | Free play | 5.7 | 1026 |
| Lee et al.[7] | 10 weeks | Not mentioned | fun health sports | 60 | 7 | Free play | 5.9 | 2478 |
| Lofrano-Prado et al.[8] | 24 weeks | 15±1 | Recreation exercise | 60 | 2 | Free play | 6 | 720 |
| Migueles et al.[9] | 20 weeks | 10±1.1 | Aerobic and muscle-bone-strength-ening activities | 90 | 3-5 | Free play | 5.9 | 2655 |
| Petty et al.[10] | 10-15 weeks | 8-11 | Low aerobic exercise | 20 | 7 | Jog slow | 5.9 | 826 |
|  |  |  | high aerobic exercise | 40 | 7 | Jog fast | 7.9 | 2212 |
| Romero-Pérez et al.[11] | 20 weeks | 10.02±0.79 | aerobic exercise + playful activities | 50 | 2 | Free play | 5.9 | 590 |
| Schranz et al.[12] | 24 weeks | 14.9±1.4 | multijoint exercises and single-joint exercises | 75 | 3 | Leg press | 2.7 | 607.5 |
| Staiano et al.[13] | 20 weeks | 15-19 | exergaming | 30-60 | 7 | Free play | 6.1 | 2562 |
| Wagener et al.[14] | 10 weeks | 14±1.66 | exergaming | 40 | 3 | Active vedio games-dance | 4.1 | 492 |
| Watson et al.[15] | 12weeks | 14.8±2.3 | aerobic and resistance exercises | 60-90 | 3 | Free play | 6 | 1620 |
| Weintraub et al.[16] | 24 weeks | 10-11 | aerobic exercise | 75 | 3-4 | Soccer-around cones | 5.6 | 1260 |
| Williams et al.[17] | 32 weeks | 10.4±0.87 | aerobic activities ang games | 40 | 7 | Free play | 5.9 | 1652 |
| Young et al.[18] | 8 weeks | 10-12 | aerobic activities | 60-70 | 7 | Free play | 5.9 | 413 |
| Yu et al.[19] | 32weeks | 9.9±0.7 | aerobic activities | 60 | 5 | Free play | 6.2 | 1860 |

^a^ Youth compendium of physical activities.

**References:**

1. CROKER H, VINER RM, NICHOLLS D, HAROUN D, CHADWICK P, EDWARDS C, WELLS J, WARDLE J: **Family-based behavioural treatment of childhood obesity in a UK national health service setting: randomized controlled trial**. *INT J OBESITY* 2012, **36**(1):16-26.

2. Daley AJ, Copeland RJ, Wright NP, Roalfe A, Wales JK: **Exercise therapy as a treatment for psychopathologic conditions in obese and morbidly obese adolescents: a randomized, controlled trial**. *PEDIATRICS* 2006, **118**(5):2126-2134.

3. Danielsen YS, Nordhus IH, Juliusson PB, Maehle M, Pallesen S: **Effect of a family-based cognitive behavioural intervention on body mass index, self-esteem and symptoms of depression in children with obesity (aged 7-13): a randomised waiting list controlled trial**. *OBES RES CLIN PRACT* 2013, **7**(2):e116-e128.

4. DeBar LL, Stevens VJ, Perrin N, Wu P, Pearson J, Yarborough BJ, Dickerson J, Lynch F: **A primary care-based, multicomponent lifestyle intervention for overweight adolescent females**. *Pediatrics (Evanston)* 2012, **129**(3):e611.

5. Goldfield GS, Kenny GP, Alberga AS, Prud'Homme D, Hadjiyannakis S, Gougeon R, Phillips P, Tulloch H, Malcolm J, Doucette S *et al*: **Effects of aerobic training, resistance training, or both on psychological health in adolescents with obesity: The HEARTY randomized controlled trial.** *J CONSULT CLIN PSYCH* 2015, **83**(6):1123-1135.

6. Heidarianpour A, Shokri E, Sadeghian E, Cheraghi F, Razavi Z: **Combined training in addition to cortisol reduction can improve the mental health of girls with precocious puberty and obesity**. *FRONT PEDIATR* 2023, **11**.

7. Lee G, Choi Y: **Effects of an obesity management mentoring program for Korean children**. *APPL NURS RES* 2016, **31**:160-164.

8. Lofrano-Prado MC, Donato Junior J, Lambertucci AC, Lambertucci RH, Malik N, Ritti-Dias RM, Correia MA, Botero JP, Prado WL: **Recreational Physical Activity Improves Adherence and Dropout in a Non-Intensive Behavioral Intervention for Adolescents With Obesity**. *RES Q EXERCISE SPORT* 2022, **93**(4):659-669.

9. Migueles JH, Cadenas-Sanchez C, Lubans DR, Henriksson P, Torres-Lopez LV, Rodriguez-Ayllon M, Plaza-Florido A, Gil-Cosano JJ, Henriksson H, Escolano-Margarit MV *et al*: **Effects of an Exercise Program on Cardiometabolic and Mental Health in Children With Overweight or Obesity: A Secondary Analysis of a Randomized Clinical Trial**. *JAMA NETW OPEN* 2023, **6**(7):e2324839.

10. Petty KH, Davis CL, Tkacz J, Young-Hyman D, Waller JL: **Exercise Effects on Depressive Symptoms and Self-Worth in Overweight Children: A Randomized Controlled Trial**. *J PEDIATR PSYCHOL* 2009, **34**(9):929-939.

11. Romero-Pérez EM, González-Bernal JJ, Soto-Cámara R, González-Santos J, Tánori-Tapia JM, Rodríguez-Fernández P, Jiménez-Barrios M, Márquez S, de Paz JA: **Influence of a Physical Exercise Program in the Anxiety and Depression in Children with Obesity**. *International Journal of Environmental Research and Public Health* 2020, **17**(13):4655.

12. Schranz N, Tomkinson G, Parletta N, Petkov J, Olds T: **Can resistance training change the strength, body composition and self-concept of overweight and obese adolescent males? A randomised controlled trial**. *BRIT J SPORT MED* 2014, **48**(20):1482-1488.

13. Staiano AE, Abraham AA, Calvert SL: **Adolescent exergame play for weight loss and psychosocial improvement: a controlled physical activity intervention**. *OBESITY* 2013, **21**(3):598-601.

14. Wagener TL, Fedele DA, Mignogna MR, Hester CN, Gillaspy SR: **Psychological effects of dance‐based group exergaming in obese adolescents**. *PEDIATR OBES* 2012, **7**(5).

15. Watson PM, McKinnon A, Santino N, Bassett-Gunter RL, Calleja M, Josse AR: **Integrating needs-supportive delivery into a laboratory-based randomised controlled trial for adolescent girls with overweight and obesity: Theoretical underpinning and 12-week psychological outcomes**. *J SPORT SCI* 2021, **39**(21):2434-2443.

16. Weintraub DL, Tirumalai EC, Haydel KF, Fujimoto M, Fulton JE, Robinson TN: **Team sports for overweight children: the Stanford Sports to Prevent Obesity Randomized Trial (SPORT)**. *Arch Pediatr Adolesc Med* 2008, **162**(3):232-237.

17. Williams CF, Bustamante EE, Waller JL, Davis CL: **Exercise effects on quality of life, mood, and self-worth in overweight children: the SMART randomized controlled trial**. *TRANSL BEHAV MED* 2019, **9**(3):451-459.

18. Moon YI, Park HR, Koo HY, Kim HS: **Effects of behavior modification on body image, depression and body fat in obese Korean elementary school children**. *YONSEI MED J* 2004, **45**(1):61.

19. Yu H, Li F, Hu Y, Li C, Yuan S, Song Y, Zheng M, Gong J, He Q: **Improving the Metabolic and Mental Health of Children with Obesity: A School-Based Nutrition Education and Physical Activity Intervention in Wuhan, China**. *NUTRIENTS* 2020, **12**(1):194.
