# Supplementary material for: Does physical activity really improve anxiety and depression in overweight or obese children and adolescents? A systematic review and meta-analysis
Source: BMC Psychiatry. 2026 Jan 16;26:139. doi: 10.1186/s12888-025-07761-9 (PMC12892821; doi:10.1186/s12888-025-07761-9)
Supplement: Supplementary file 1 — Supplementary Material 1 [file 12888_2025_7761_MOESM1_ESM.zip › Appendix/Additional file 2 Boolean search strings.docx]

**Additional file 2** Boolean search strings

### PubMed

| Items | Search Strategy | Search Results |
| --- | --- | --- |
| #1 | "adolescent"[MeSH Terms] | 2,350,107 |
| #2 | "child"[Title/Abstract] OR "young"[Title/Abstract] OR "boy"[Title/Abstract] OR "girl"[Title/Abstract] OR "teen"[Title/Abstract] OR "kid"[Title/Abstract] OR " school age "[Title/Abstract] OR " schoolchild "[Title/Abstract] OR "youth "[Title/Abstract] OR "juvenile"[Title/Abstract] | 1,430,244 |
| #3 | "exercise"[MeSH Terms] | 276,100 |
| #4 | "motor activity"[Title/Abstract] OR "physical activity"[Title/Abstract] OR "resistance training"[Title/Abstract] OR "aerobic exercise"[Title/Abstract] OR "acute exercise"[Title/Abstract] OR "strength training"[Title/Abstract] OR "endurance training"[Title/Abstract] OR "Sports"[Title/Abstract] OR "yoga"[Title/Abstract] OR "basketball"[Title/Abstract] OR "soccer"[Title/Abstract] | 304,377 |
| #5 | "obesity"[MeSH Terms] | 281,001 |
| #6 | "Overweight"[Title/Abstract] OR"Obese"[Title/Abstract] OR "Adiposity"[Title/Abstract] | 239,319 |
| #7 | "depression"[MeSH Terms] | 277,211 |
| #8 | "psychological symptoms"[Title/Abstract] OR "psychopathologic conditions"[Title/Abstract] OR "self-esteem"[Title/Abstract] OR "depressive disorder"[Title/Abstract] OR "anxiety"[Title/Abstract] OR (self-worth[Title/Abstract]) | 371,415 |
| #9 | #1 or #2 | 3,340,662 |
| #10 | #3 or #4 | 471,408 |
| #11 | #5 or #6 | 385,723 |
| #12 | #7 or #8 | 555,260 |
| #13 | #9 and #10 and #11 and #12 | 509 |

### Embase

| Items | Search Terms | Search Results |
| --- | --- | --- |
| #1 | 'adolescent'/exp OR 'adolescent' OR 'child'/exp OR 'child' OR 'young' OR 'boy'/exp OR 'boy' OR 'girl'/exp OR 'girl' OR 'teen' OR 'kid' OR 'school age'/exp OR 'school age' OR 'schoolchild'/exp OR 'schoolchild' OR 'youth'/exp OR 'youth' OR 'juvenile'/exp OR 'juvenile' | 6,466,297 |
| #2 | 'exercise'/exp OR 'exercise' OR 'motor activity'/exp OR 'motor activity' OR 'physical activity'/exp OR 'physical activity' OR 'exercise interventions' OR 'resistance training'/exp OR 'resistance training' OR 'aerobic exercise'/exp OR 'aerobic exercise' OR 'acute exercise'/exp OR 'acute exercise' OR 'strength training'/exp OR 'strength training' OR 'endurance training'/exp OR 'endurance training' OR 'sports'/exp OR 'sports' OR 'basketball'/exp OR 'basketball' OR 'soccer'/exp OR 'soccer' OR 'yoga'/exp OR 'yoga' | 2,061,331 |
| #3 | 'overweight'/exp OR 'overweight' OR 'obese' OR 'adiposity'/exp OR 'adiposity' OR 'obesity'/exp OR 'obesity' | 937,993 |
| #4 | 'psychology mental disorders' OR 'psychological symptoms' OR 'psychopathologic conditions' OR 'self-esteem'/exp OR 'self-esteem' OR 'depressive disorder'/exp OR 'depressive disorder' OR 'anxiety'/exp OR 'anxiety' OR 'depression'/exp OR 'depression' OR 'self-worth' | 1,324,397 |
| #5 | #1 AND #2 AND #3 AND #4 AND [randomized controlled trial]/lim | 253 |

### Cochrane Library

| Items | Search Terms | Search Results |
| --- | --- | --- |
| #1 | MeSH descriptor: [Adolescent] explode all trees | 135760 |
| #2 | (Child or young or boy or girl or teen or kid or school age or schoolchild or youth or juvenile):ti,ab,kw | 336311 |
| #3 | #1 or #2 | 384951 |
| #4 | MeSH descriptor: [Exercise] explode all trees | 39411 |
| #5 | (Motor activity or physical activity or Resistance Training or Train or Aerobic exercise or acute exercise or strength training or Endurance Training or Sports or meditation or yoga or basketball or soccer):ti,ab,kw | 258325 |
| #6 | #4 or #5 | 267676 |
| #7 | MeSH descriptor: [Obesity] explode all trees | 21557 |
| #8 | (Overweight or Obese or Adiposity):ti,ab,kw | 68041 |
| #9 | #7 or #8 | 68140 |
| #10 | MeSH descriptor: [Depression] explode all trees | 18820 |
| #11 | (Psychology Mental Disorders or psychological symptoms or psychopathologic conditions or self-esteem or Depressive Disorder or Anxiety):ti,ab,kw | 141367 |
| #12 | #10 or #11 | 145451 |
| #13 | #3 and #6 and #9 and #12 | 481 |

### CNKI：

| Search Terms | Search Results |
| --- | --- |
| (SU="儿童青少年" OR SU="儿童" OR SU="青少年" OR SU="小学生" OR SU="初中生" OR SU="高中生" OR SU="学生" OR SU="男生" OR SU="女生") AND (SU="心理健康" OR SU="心理亚健康" OR SU="身心健康" OR SU="焦虑" OR SU="抑郁" OR SU="情绪" OR SU="自尊" OR SU="自我价值") AND (SU="身体活动" OR SU="运动" OR SU="训练" OR SU="锻炼" OR SU="体力活动" OR SU="户外运动" OR SU="步行" OR SU="体育活动" OR SU="有氧运动" OR SU="抗阻训练" OR SU="力量训练") AND (SU="肥胖" OR SU="超重") | 290 |

### WanFang：

| Search Terms | Search Results |
| --- | --- |
| (主题:"儿童青少年" or 主题: "儿童" or 主题:"青少年" or 主题:"小学生" or 主题:"初中生" or 主题:"高中生" or 主题:"学生" or 主题:"男生" or 主题:"女生") and (主题:"心理健康" or 主题:"心理亚健康" or 主题:"身心健康" or 主题:"焦虑" or 主题:"抑郁" or 主题:"情绪" or 主题:"自尊" or 主题:"自我价值") and (主题:"身体活动" or 主题:"运动" or 主题:"训练" or 主题:"锻炼" or 主题:"体力活动" or 主题:"户外运动" or 主题:"步行" or 主题:"体力活动" or 主题:"有氧运动" or 主题:"抗阻训练" or 主题:"力量训练") and (主题:"肥胖" or 主题:"超重") | 1184 |
