# Supplementary material for: Does physical activity really improve anxiety and depression in overweight or obese children and adolescents? A systematic review and meta-analysis
Source: BMC Psychiatry. 2026 Jan 16;26:139. doi: 10.1186/s12888-025-07761-9 (PMC12892821; doi:10.1186/s12888-025-07761-9)
Supplement: Supplementary file 1 — Supplementary Material 1 [file 12888_2025_7761_MOESM1_ESM.zip › Appendix/Additional file 20 Sensitivity Analysis of the Correlation Coefficient (Corr).docx]

**Additional file 20** Sensitivity Analysis of the Correlation Coefficient (Corr)

| **Outcome** | **Corr** | **Hedges'g (SMD)** | **95% CI** | ***I²* (%)** |
| --- | --- | --- | --- | --- |
| Anxiety | 0.3 | -0.84 | [-1.63, -0.04] | 93 |
|  | **0.5** | **-0.98** | **[-1.90, -0.05]** | 95 |
|  | 0.7 | -1.21 | [-2.37, -0.06] | 97 |
| Depression | 0.3 | -0.13 | [-0.22, -0.05] | 0 |
|  | **0.5** | **-0.15** | **[-0.25, -0.05]** | 0 |
|  | 0.7 | -0.05 | [-0.11, 0.01] | 0 |
| Slef-esteem | 0.3 | 0.19 | [0.03, 0.35] | 0 |
|  | **0.5** | **0.19** | **[0.03, 0.35]** | 0 |
|  | 0.7 | 0.37 | [0.09, 0.64] | 59 |
| Self-worth | 0.3 | 0.29 | [0.16, 0.41] | 0 |
|  | **0.5** | **0.34** | **[0.19, 0.49]** | 2 |
|  | 0.7 | 0.47 | [0.26, 0.67] | 33 |

Corr = correlation coefficient; Hedges’ g (SMD) = Hedges-corrected standardized mean difference; 95% CI = 95% confidence interval; *I²* (%) = proportion of heterogeneity.
