# Supplementary material for: Does physical activity really improve anxiety and depression in overweight or obese children and adolescents? A systematic review and meta-analysis
Source: BMC Psychiatry. 2026 Jan 16;26:139. doi: 10.1186/s12888-025-07761-9 (PMC12892821; doi:10.1186/s12888-025-07761-9)
Supplement: Supplementary file 1 — Supplementary Material 1 [file 12888_2025_7761_MOESM1_ESM.zip › Appendix/Additional file 21 Sensitivity Analysis Including Only Studies with Physical Activity as the Sole Variable.docx]

| **Outcome** | **k** | **Hedges'g (SMD)** | **95% CI** | **P-value** |
| --- | --- | --- | --- | --- |
| Depression | 13 | -0.1354 | -0.2657 to -0.0052 | 0.0416^*^ |
| Self-esteem | 8 | 0.1444 | -0.0488 to 0.3375 | 0.1429 |
| Self-worth | 9 | 0.2648 | 0.1071 to 0.4225 | 0.0010^**^ |

**Additional file 21** Sensitivity Analysis Including Only Studies with Physical Activity as the Sole Variable

k indicates the number of included studies. SMD (Hedges’ g) denotes the standardized mean difference. 95% CI represents the 95% confidence interval of the pooled effect. *P* < 0.05 was considered statistically significant, and *P* < 0.01 indicates high statistical significance. **P*＜0.05，***P*＜0.01
