# Supplementary material for: Does physical activity really improve anxiety and depression in overweight or obese children and adolescents? A systematic review and meta-analysis
Source: BMC Psychiatry. 2026 Jan 16;26:139. doi: 10.1186/s12888-025-07761-9 (PMC12892821; doi:10.1186/s12888-025-07761-9)
Supplement: Supplementary file 1 — Supplementary Material 1 [file 12888_2025_7761_MOESM1_ESM.zip › Appendix/Additional file 22 Funnel plot.docx]

Additional file 22 Funnel plot

#### Funnel plot for anxiety (n=5 studies)


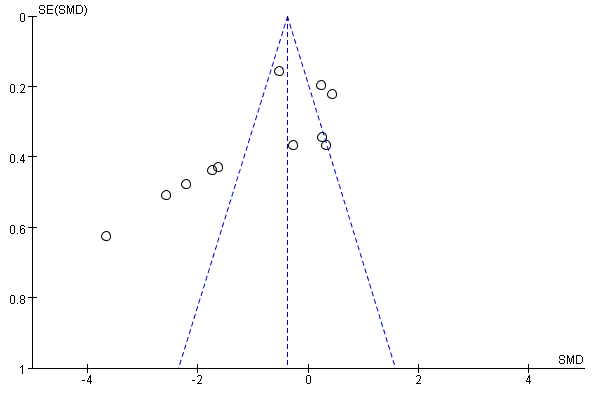


#### Funnel plot for depression (n=14 studies)


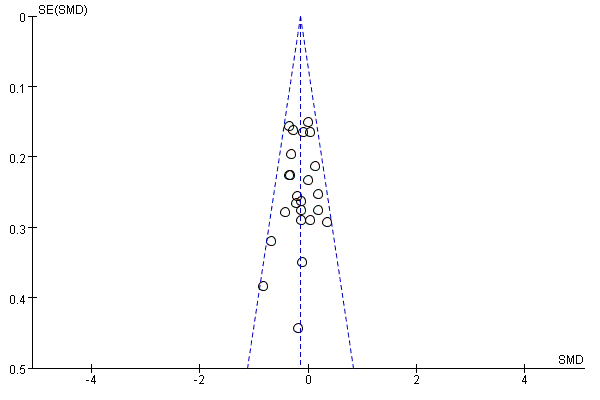


#### Funnel plot for self-esteem (n=9 studies)


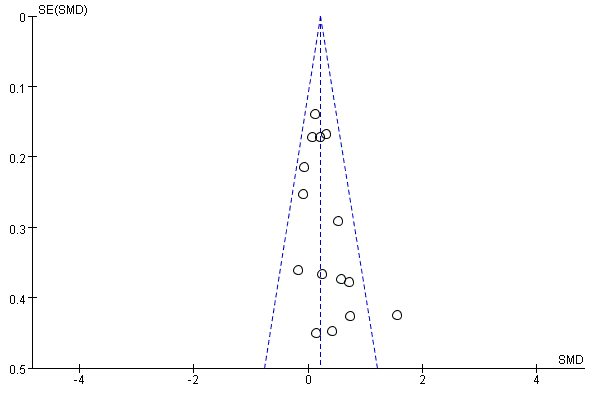


#### Funnel plot for self-worth (n=7 studies)


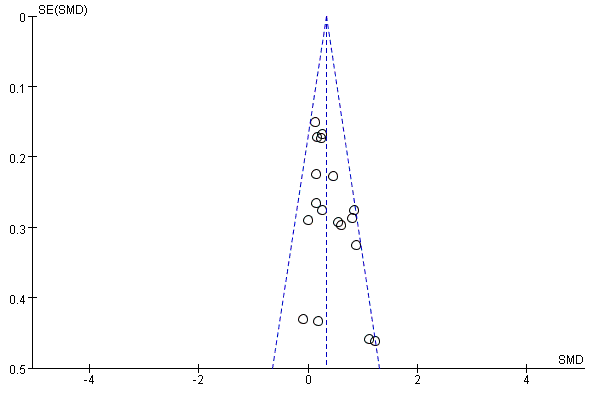


Each circle represents an individual study. The plot shows the relationship between standardized mean differences (SMD) and their standard errors (SE). The dashed lines represent the 95% confidence limits, and symmetry around the vertical line at SMD = 0 indicates the absence of publication bias.
