# Supplementary material for: Does physical activity really improve anxiety and depression in overweight or obese children and adolescents? A systematic review and meta-analysis
Source: BMC Psychiatry. 2026 Jan 16;26:139. doi: 10.1186/s12888-025-07761-9 (PMC12892821; doi:10.1186/s12888-025-07761-9)
Supplement: Supplementary file 1 — Supplementary Material 1 [file 12888_2025_7761_MOESM1_ESM.zip › Appendix/Additional file 23 Trim-and-Fill funnel plot and Egger's test.docx]

**Additional file 23** Trim-and-Fill funnel plot and Egger's test

| **Summary of Publication Bias Analyses for Each Outcome** | | | | | | |
| --- | --- | --- | --- | --- | --- | --- |
| **Outcomes** | **Egger** | **No. of Imputed Studies (k₀)** | **Original Effect** | **Original 95% CI** | **Adjusted Effect** | **Adjusted 95% CI** |
| Anxiety | 0.04* | 0 | -0.98 | -1.63 to 0.62 | -0.98 | -1.63 to 0.62 |
| Depression | 0.69 | 0 | -0.15 | -0.17 to 0.04 | -0.15 | -0.17 to 0.04 |
| Self-esteem | 0.07 | 4 | 0.19 | 0.07 to 0.31 | 0.12 | 0.01 to 0.24 |
| Self-worth | 0.02* | 2 | 0.35 | 0.22 to 0.48 | 0.32 | 0.19 to 0.45 |
| Egger’s *p* < 0.05 indicates potential publication bias.  k₀ = number of studies imputed using the Trim-and-Fill method  The Trim-and-Fill method by Duval and Tweedie was used to estimate the number of missing studies and adjust the pooled effect size.  95% CI = 95% confidence interval.  *P* values < 0.05 were considered statistically significant. **P*＜0.05 | | | | | | |
