# Supplementary material for: Does physical activity really improve anxiety and depression in overweight or obese children and adolescents? A systematic review and meta-analysis
Source: BMC Psychiatry. 2026 Jan 16;26:139. doi: 10.1186/s12888-025-07761-9 (PMC12892821; doi:10.1186/s12888-025-07761-9)
Supplement: Supplementary file 1 — Supplementary Material 1 [file 12888_2025_7761_MOESM1_ESM.zip › Appendix/Additional file 24 GRADE summary of findings for 4 outcomes.docx]

**Additional file 24** GRADE summary of findings for 4 outcomes

| **Outcome** | **Initial rating** | **Risk of bias** | **Inconsistency** | **Indirectness** | **Imprecision** | **Publication bias** | **Quality of the**  **evidence** |
| --- | --- | --- | --- | --- | --- | --- | --- |
| Anxiety | High | No serious concern | Serious concern ^b^ | No serious concern | No serious concern | Serious concern ^c^ | Low |
| Depression | High | Serious concern ^a^ | No serious concern | No serious concern | No serious concern | No serious concern | Moderate |
| Self-esteem | High | Serious concern ^a^ | No serious concern | No serious concern | No serious concern | No serious concern | Moderate |
| Self-worth | High | Serious concern ^a^ | No serious concern | No serious concern | No serious concern | Serious concern ^c^ | Low |

a: Downgraded one level due to some concerns about risk of bias

b: Downgraded one level due to unexplained heterogeneity

c: Downgraded one level due to suspected publication bias
