# Supplementary material for: Does physical activity really improve anxiety and depression in overweight or obese children and adolescents? A systematic review and meta-analysis
Source: BMC Psychiatry. 2026 Jan 16;26:139. doi: 10.1186/s12888-025-07761-9 (PMC12892821; doi:10.1186/s12888-025-07761-9)
Supplement: Supplementary file 1 — Supplementary Material 1 [file 12888_2025_7761_MOESM1_ESM.zip › Appendix/Additional file 4 Excluded studies and reasons for exclusion.docx]

**Additional file 4** Excluded studies and reasons for exclusion

First search (From the inception of each database until January 1, 2024)

| N | First Author（Year） | Title | Reason for exclusion |
| --- | --- | --- | --- |
| 1 | Braet(2004)[1] | Inpatient Treatment for Children With Obesity: Weight Loss, Psychological Well-being, and Eating Behavior | Wrong study design |
| 2 | Brennan(2012)[2] | Treatment Acceptability and Psychosocial Outcomes of a Randomised Controlled Trial of a Cognitive Behavioural Lifestyle Intervention for Overweight and Obese Adolescents | Wrong study design |
| 3 | Christison(2012)[3] | Exergaming for Health: A Community-Based Pediatric Weight Management Program Using Active Video Gaming | Wrong study design |
| 4 | Duman(2016)[4] | The Role of Active Video-Accompanied Exercises in Improvement of the Obese State in Children: A Prospective Study from Turkey | Wrong study design |
| 5 | Emmanouil(2018)[5] | The effectiveness of a health promotion and stress-management intervention program in a sample of obese children and adolescents | Wrong study design |
| 6 | Fidelix(2019)[6] | Aerobic Training Performed at Ventilatory Threshold Improves Psychological Outcomes in Adolescents With Obesity | Wrong study design |
| 7 | Gunnarsdottir(2012)[7] | Childhood obesity and co-morbid problems: effects of Epstein’s family-based behavioural treatment in an Icelandic sample | Wrong study design |
| 8 | Jelalian(2019)[8] | Cognitive-Behavioral Therapy Plus Healthy Lifestyle Enhancement for Depressed, Overweight/ Obese Adolescents: Results of a Pilot Trial | Wrong study design |
| 9 | Levine(2001)[9] | Is Family-Based Behavioral Weight Control Appropriate for Severe Pediatric Obesity? | Wrong study design |
| 10 | Lofrano-Prado(2009)[10] | Quality of life in Brazilian obese adolescents: effects of a long-term multidisciplinary lifestyle therapy | Wrong study design |
| 11 | Nobles(2016)[11] | Psychosocial Interventions in the Treatment of Severe Adolescent Obesity: The SHINE Program | Wrong study design |
| 12 | Quinlan(2009)[12] | Psychosocial outcomes in a weight loss camp for overweight youth | Wrong study design |
| 13 | Sacher(2005)[13] | Assessing the acceptability and feasibility of the MEND Programme in a small group of obese 7–11year-old children | Wrong study design |
| 14 | Tronieri(2019)[14] | A pilot study of acceptance-based behavioural weight loss for adolescents with obesity | Wrong study design |
| 15 | Chen(2024)[15] | Effect of physical activity on anxiety, depression and obesity index in children and adolescents with obesity: A meta-analysis | Systematic review |
| N | First Author（Year） | Title | Reason for exclusion |
| 16 | Lowry(2007)[16] | The Effects of Weight Management Programs on Self-Esteem in Pediatric Overweight Populations | Systematic review |
| 17 | Latino(2023)[17] | Classroom-Based Physical Activity as a Means to Improve Self-Efficacy and Academic Achievement among Normal-Weight and Overweight Youth | Wrong participants |
| 18 | Morgan(2010)[18] | Improving physical self-perception in adolescent boys from disadvantaged schools: psychological outcomes from the Physical Activity Leaders randomized controlled trial | Wrong participants |
| 19 | Velez(2010)[19] | THE IMPACT OF A 12-WEEK RESISTANCE TRAINING PROGRAM ON STRENGTH, BODY COMPOSITION, AND SELF-CONCEPT OF HISPANIC ADOLESCENTS | Wrong participants |
| 20 | Neumark-Sztainer(2003)[20] | New Moves: a school-based obesity prevention program for adolescent girls | Ineligible study data |
| 21 | Pott(2010)[21] | Course of Depressive Symptoms in Overweight Youth Participating in a Lifestyle Intervention: Associations With Weight Reduction | Ineligible study data |
| 22 | Alberga(2023)[22] | Effects of the HEARTY exercise randomized controlled trial on eating behaviors in adolescents with obesity | Wrong outcomes |
| 23 | Haapala(2024)[23] | Which indices of cardiorespiratory fitness are more strongly associated with brain health in children with overweight/obesity? | Wrong outcomes |
| 24 | Mora-Gonzalez(2023)[24] | The effects of an exercise intervention on neuroelectric activity and executive function in children with overweight/obesity: The ActiveBrains randomized controlled trial | Wrong outcomes |
| 25 | Panagiotopoulos(2011)[25] | The Centre for Healthy Weights—Shapedown BC: A Family-Centered, Multidisciplinary Program that Reduces Weight Gain in Obese Children over the Short-Term | Wrong outcomes |
| 26 | Rodriguez-Ayllon(2023)[26] | The effects of a 20-week exercise program on blood-circulating biomarkers related to brain health in overweight or obese children: The ActiveBrains project | Wrong outcomes |
| 27 | Stella(2005)[27] | Effects of type of physical exercise and leisure activities on the depression scores of obese Brazilian adolescent girls | Wrong outcomes |
| 28 | Srivastav(2021)[28] | Structured, multifactorial randomised controlled intervention to investigate physical activity levels, body composition and diet in obese and overweight adolescents | Wrong outcomes |

Second search (Between January 1, 2024, and March 1, 2025)

| N | First Author（Year） | Title | Reason for exclusion |
| --- | --- | --- | --- |
| 1 | Sepúlveda(2024)[29] | [Testing a Family System-based Intervention (ENTREN-F Programme) for a paediatric obesity sample by a Randomized Controlled Trial](https://www.cochranelibrary.com/central/doi/10.1002/central/CN-02760411/full) | Wrong study design |
| 2 | Weiner(2024)[30] | [Engagement, Acceptability, and Effectiveness of the Self-Care and Coach-Supported Versions of the Vira Digital Behavior Change Platform Among Young Adults at Risk for Depression and Obesity: pilot Randomized Controlled Trial](https://www.cochranelibrary.com/central/doi/10.1002/central/CN-02751377/full) | Wrong participants |
| 3 | Evans(2025)[31] | [Mechanisms and outcomes of a very low intensity intervention to improve parental acknowledgement and understanding of childhood overweight/obesity, embedded in the National Child Measurement Programme: a sub-study within a large cluster Randomized Controlled Trial (MapMe2)](https://www.cochranelibrary.com/central/doi/10.1002/central/CN-02810050/full) | Wrong outcomes |
| 4 | Van Royen(2023)[32] | [Treatment Effects on Psychophysiological Stress Responses in Youth With Obesity](https://www.cochranelibrary.com/central/doi/10.1002/central/CN-02634624/full) | Wrong outcomes |
| 5 | Adem SÜMEN(2025) | [Adolescents' Health Literacy, Physical Activity and Healthy Eating Self-efficacy Levels](https://www.cochranelibrary.com/central/doi/10.1002/central/CN-02808938/full) | Ongoing clinical studies |
| 6 | Cairo University(2025) | [Effect of Aerobic Exercise Program on Blood Glucose Level and Quality of Life in Pre-diabetic Overweight/Obese Adolescents](https://www.cochranelibrary.com/central/doi/10.1002/central/CN-02800046/full) | Ongoing clinical studies |
| 7 | Holbaek Sygehus(2025) | [Holb k Obesity Treatment (HOT) versus Conventional Obesity Treatment (COT) in children with obesity - An investigatorinitiated, multi-centre RCT with blinded outcome assessment](https://www.cochranelibrary.com/central/doi/10.1002/central/CN-02710420/full) | Ongoing clinical studies |
| 8 | Fatemeh Sadeghi(2025) | [Investigating the effect of virtual sports training on self-concept and self-esteem of obese girls](https://www.cochranelibrary.com/central/doi/10.1002/central/CN-02626422/full) | Ongoing clinical studies |

Ongoing clinical trials cannot be cited in the literature.

**References:**

1. Braet C, Tanghe A, Decaluwé V, Moens E, Rosseel Y: **Inpatient treatment for children with obesity: weight loss, psychological well-being, and eating behavior**. *J PEDIATR PSYCHOL* 2004, **29**(7):519-529.

2. Brennan L, Wilks R, Walkley J, Fraser SF, Greenway K: **Treatment Acceptability and Psychosocial Outcomes of a Randomised Controlled Trial of a Cognitive Behavioural Lifestyle Intervention for Overweight and Obese Adolescents**. *BEHAV CHANGE* 2012, **29**(1):36-62.

3. Christison A, Khan HA: **Exergaming for health: a community-based pediatric weight management program using active video gaming**. *CLIN PEDIATR* 2012, **51**(4):382-388.

4. Duman F, Kokaçya MH, Doğru E, Katayıfcı N, Canbay Ö, Aman F: **The Role of Active Video-Accompanied Exercises in Improvement of the Obese State in Children: A Prospective Study from Turkey**. *J CLIN RES PEDIATR E* 2016, **8**(3):334-340.

5. Emmanouil CC, Pervanidou P, Charmandari E, Darviri C, Chrousos GP: **The effectiveness of a health promotion and stress-management intervention program in a sample of obese children and adolescents**. *HORM-INT J ENDOCRINO* 2018, **17**(3):405-413.

6. Fidelix Y, Lofrano-Prado MC, Fortes LS, Hill JO, Caldwell AE, Botero JP, Do PW: **Aerobic Training Performed at Ventilatory Threshold Improves Psychological Outcomes in Adolescents With Obesity**. *J PHYS ACT HEALTH* 2019, **16**(10):851-856.

7. Gunnarsdottir T, Njardvik U, Olafsdottir AS, Craighead L, Bjarnason R: **Childhood obesity and co-morbid problems: effects of Epstein's family-based behavioural treatment in an Icelandic sample**. *J EVAL CLIN PRACT* 2012, **18**(2):465-472.

8. Jelalian E, Jandasek B, Wolff JC, Seaboyer LM, Jones RN, Spirito A: **Cognitive-Behavioral Therapy Plus Healthy Lifestyle Enhancement for Depressed, Overweight/Obese Adolescents: Results of a Pilot Trial**. *J CLIN CHILD ADOLESC* 2019, **48**(sup1):S24-S33.

9. Levine MD, Ringham RM, Kalarchian MA, Wisniewski L, Marcus MD: **Is family-based behavioral weight control appropriate for severe pediatric obesity?** *INT J EAT DISORDER* 2001, **30**(3):318-328.

10. Lofrano-Prado MC, Antunes HK, Do PW, de Piano A, Caranti DA, Tock L, Carnier J, Tufik S, de Mello MT, Dâmaso AR: **Quality of life in Brazilian obese adolescents: effects of a long-term multidisciplinary lifestyle therapy**. *HEALTH QUAL LIFE OUT* 2009, **7**:61.

11. Nobles J, Radley D, Dimitri P, Sharman K: **Psychosocial Interventions in the Treatment of Severe Adolescent Obesity: The SHINE Program**. *J ADOLESCENT HEALTH* 2016, **59**(5):523-529.

12. Quinlan NP, Kolotkin RL, Fuemmeler BF, Costanzo PR: **Psychosocial outcomes in a weight loss camp for overweight youth**. *Int J Pediatr Obes* 2009, **4**(3):134-142.

13. Sacher PM, Chadwick P, Wells JC, Williams JE, Cole TJ, Lawson MS: **Assessing the acceptability and feasibility of the MEND Programme in a small group of obese 7-11-year-old children**. *J HUM NUTR DIET* 2005, **18**(1):3-5.

14. Tronieri JS, Wadden TA, Leonard SM, Berkowitz RI: **A pilot study of acceptance-based behavioural weight loss for adolescents with obesity**. *BEHAV COGN PSYCHOTH* 2019, **47**(6):686-696.

15. Chen L, Liu Q, Xu F, Wang F, Luo S, An X, Chen J, Tang N, Jiang X, Liang X: **Effect of physical activity on anxiety, depression and obesity index in children and adolescents with obesity: A meta-analysis**. *J AFFECT DISORDERS* 2024, **354**:275-285.

16. Lowry KW, Sallinen BJ, Janicke DM: **The effects of weight management programs on self-esteem in pediatric overweight populations**. *J PEDIATR PSYCHOL* 2007, **32**(10):1179-1195.

17. Latino F, Tafuri F, Saraiello E, Tafuri D: **Classroom-Based Physical Activity as a Means to Improve Self-Efficacy and Academic Achievement among Normal-Weight and Overweight Youth**. *NUTRIENTS* 2023, **15**(9).

18. Morgan PJ, Saunders KL, Lubans DR: **Improving physical self-perception in adolescent boys from disadvantaged schools: psychological outcomes from the Physical Activity Leaders randomized controlled trial**. *PEDIATR OBES* 2012, **7**(3):e27-e32.

19. Velez A, Golem DL, Arent SM: **The impact of a 12-week resistance training program on strength, body composition, and self-concept of Hispanic adolescents**. *J STRENGTH COND RES* 2010, **24**(4):1065-1073.

20. Neumark-Sztainer D, Story M, Hannan PJ, Rex J: **New Moves: a school-based obesity prevention program for adolescent girls**. *PREV MED* 2003, **37**(1):41-51.

21. Pott W, Albayrak O, Hebebrand J, Pauli-Pott U: **Course of depressive symptoms in overweight youth participating in a lifestyle intervention: associations with weight reduction**. *J DEV BEHAV PEDIATR* 2010, **31**(8):635-640.

22. Alberga AS, Edache IY, Sigal RJ, von Ranson KM, Russell-Mayhew S, Kenny GP, Doucette S, Prud'Homme D, Hadjiyannakis S, Cameron JD *et al*: **Effects of the HEARTY exercise randomized controlled trial on eating behaviors in adolescents with obesity**. *OBES SCI PRACT* 2023, **9**(2):158-171.

23. Haapala EA, Lubans DR, Jaakkola T, Barker AR, Plaza-Florido A, Gracia-Marco L, Solis-Urra P, Cadenas-Sanchez C, Esteban-Cornejo I, Ortega FB: **Which indices of cardiorespiratory fitness are more strongly associated with brain health in children with overweight/obesity?** *SCAND J MED SCI SPOR* 2024, **34**(1):e14549.

24. Mora-Gonzalez J, Esteban-Cornejo I, Solis-Urra P, Rodriguez-Ayllon M, Cadenas-Sanchez C, Hillman CH, Kramer AF, Catena A, Ortega FB: **The effects of an exercise intervention on neuroelectric activity and executive function in children with overweight/obesity: The ActiveBrains randomized controlled trial**. *SCAND J MED SCI SPOR* 2024, **34**(1):e14486.

25. Panagiotopoulos C, Ronsley R, Al-Dubayee M, Brant R, Kuzeljevic B, Rurak E, Cristall A, Marks G, Sneddon P, Hinchliffe M *et al*: **The centre for healthy weights--shapedown BC: a family-centered, multidisciplinary program that reduces weight gain in obese children over the short-term**. *INT J ENV RES PUB HE* 2011, **8**(12):4662-4678.

26. Rodriguez-Ayllon M, Plaza-Florido A, Mendez-Gutierrez A, Altmäe S, Solis-Urra P, Aguilera CM, Catena A, Ortega FB, Esteban-Cornejo I: **The effects of a 20-week exercise program on blood-circulating biomarkers related to brain health in overweight or obese children: The ActiveBrains project**. *J SPORT HEALTH SCI* 2023, **12**(2):175-185.

27. Stella SG, Vilar AP, Lacroix C, Fisberg M, Santos RF, Mello MT, Tufik S: **Effects of type of physical exercise and leisure activities on the depression scores of obese Brazilian adolescent girls**. *BRAZ J MED BIOL RES* 2005, **38**(11):1683-1689.

28. Srivastav P, K V, Bhat VH, Broadbent S: **Structured, multifactorial randomised controlled intervention to investigate physical activity levels, body composition and diet in obese and overweight adolescents**. *BMJ OPEN* 2021, **11**(3):e044895.

29. Sepúlveda AR, Rojo M, Lacruz T, Solano S, Graell M, Veiga ÓL: **Testing a family system-based intervention (ENTREN-F Programme) for a paediatric obesity sample by a randomized controlled trial**. *APPETITE* 2024, **203**:107696.

30. Weiner LS, Crowley RN, Sheeber LB, Koegler FH, Davis JF, Wells M, Funkhouser CJ, Auerbach RP, Allen NB: **Engagement, Acceptability, and Effectiveness of the Self-Care and Coach-Supported Versions of the Vira Digital Behavior Change Platform Among Young Adults at Risk for Depression and Obesity: Pilot Randomized Controlled Trial**. *JMIR MENT HEALTH* 2024, **11**:e51366.

31. Evans EH, Jones CM, Adamson A, Jones AR, Basterfield L, Greca J, Sermin-Reed L, Patterson M, McSweeney L, Dhami R *et al*: **Mechanisms and outcomes of a very low intensity intervention to improve parental acknowledgement and understanding of childhood overweight/obesity, embedded in the National Child Measurement Programme: A sub-study within a large cluster Randomized Controlled Trial (MapMe2)**. *BRIT J HEALTH PSYCH* 2025, **30**(1):e12784.

32. Van Royen A, Verbiest I, Goemaere H, Debeuf T, Michels N, Verbeken S, Braet C: **Treatment Effects on Psychophysiological Stress Responses in Youth With Obesity**. *PSYCHOSOM MED* 2024, **86**(1):11-19.
