# Supplementary material for: Does physical activity really improve anxiety and depression in overweight or obese children and adolescents? A systematic review and meta-analysis
Source: BMC Psychiatry. 2026 Jan 16;26:139. doi: 10.1186/s12888-025-07761-9 (PMC12892821; doi:10.1186/s12888-025-07761-9)
Supplement: Supplementary file 1 — Supplementary Material 1 [file 12888_2025_7761_MOESM1_ESM.zip › Appendix/Additional file 5 Additional characteristics of the included studies.docx]

**Additional file 5** Additional characteristics of the included studies

| Author | Year | Country | Target population | Age | | Sample | | Intervention measure | | Intervention timouthse | | Outcome indicator |
| --- | --- | --- | --- | --- | --- | --- | --- | --- | --- | --- | --- | --- |
|  |  |  |  | experimental group | control group | experimental group（male/female） | control group **（male/female）** | experimental group | control group | experimental group | control group |  |
| Croker et al.[1] | 2011 | UK | Obese | 10.8 ± 1.6 | 9.8 ± 1.4 | 33 | 30 | mode of motion: structured activity  exercise time: 60 mins  exercise frequency:per day | mode of motion: daily activities | 24 weeks | 24 weeks | 2,3 |
| Daley et al.[2] | 2006 | UK | Obese | 13.1 | 13.1 | 49 | 26 | mode of motion: aerobic exercise and light body-conditioning or stretching exercises  exercise time:30 mins  exercise frequency: three times per week  exercise intensity: ＜59% of HR reserve | mode of motion: daily activities | 8 weeks | 8 weeks | 2,4 |
| Danielsen et al.[3] | 2013 | Norway | Obese | 10.68 ± 1.24 | 10.68 ± 1.24 | 22 | 20 | mode of motion: aerobic exercise  exercise time: 30 mins  exercise frequency: seven times per week | mode of motion: daily activities | 12 weeks | 12 weeks | 2,4 |
| DeBar et al.[4] | 2012 | America | Overweight girls | 14.12 ± 1.48 | 14.03 ± 1.50 | 104 | 102 | mode of motion: Yoga,Dance Video Games and aerobic training  exercise time: 30-60 mins  exercise frequency: five times per week | mode of motion: daily activities | 24 weeks | 24 weeks | 3 |
| Goldfield et al.[5] | 2015 | Canada | Obese | 14-17 | 15.6 ± 1.3 | 228 | 76 | mode of motion:aerobic exercise and resistance training  exercise time:20-45 mins  exercise frequency: four times per week  exercise intensity: 65-85%HRmax or 8RM of repetition maximum | mode of motion: daily activities | 22 weeks | 22 weeks | 2,3,4 |
| Heidarianpour et al.[6] | 2023 | Iran | central precocious puberty and (CPP)Obese girls | 8.51 ± 0.44 | 8.35 ± 0.59 | 15 | 15 | mode of motion: combined aerobic and resistance training  exercise time: 60 mins  exercise frequency: three times per week  exercise intensity: 55%–65% of individual maximal cardiorespiratory fitness | mode of motion: daily activities | 12 weeks | 12 weeks | 1,2 |
| Lee et al.[7] | 2016 | Korean | Overweight/Obese | Not mentioned | Not mentioned | 15 | 15 | mode of motion: fun health sports  exercise frequency: per day | mode of motion: daily activities | 10 weeks | 10 weeks | 3 |
| Lofrano-Prado et al.[8] | 2022 | Brazil | Obese | 15±1 | 15±1 | 37 | 37 | mode of motion: Recreation exercise  exercise time: 60 mins  exercise frequency: two times per week | mode of motion: daily activities | 24 weeks | 24 weeks | 2 |
| Migueles et al.[9] | 2023 | Spain | Overweight/Obese | 10±1.1 | 10.1±1.1 | 47 | 45 | mode of motion: aerobic and muscle-bone–strengthening activities  exercise time: 90 mins  exercise frequency: three~five times per week  exercise intensity: ＞80%HRmax | mode of motion: daily activities | 20 weeks | 20 weeks | 1,2,3 |
| Petty et al.[10] | 2009 | Korea | Overweight | 8-11 | 9.4 ± 1.1 | 139 | 68 | mode of motion: low/high aerobic exercise  exercise time: 20-40 mins  exercise frequency: seven times per week | mode of motion: daily activities | 10-15 weeks | 10-15 weeks | 2,4 |
| Author | Year | Country | target population | Age | | Sample | | Intervention measure | | Intervention timouthse | | Outcome indicator |
|  |  |  |  | experimental group | control group | experimental group（male/female） | control group **（male/female）** | experimental group | control group | experimental group | control group |  |
| Romero-Pérez et al.[11] | 2020 | Mexico | Obese | 10.02 ± 0.79 | 10.02 ± 0.79 | 54  (24/30) | 51  (21/30) | mode of motion: aerobic exercise and playful activities  exercise time: 50 mins  exercise frequency: two times per week | mode of motion: daily activities | 20 weeks | 20 weeks | 1,2 |
| Schranz et al.[12] | 2013 | Australia | Overweight/Obese boys | 14.9 ± 1.4 | 15.1 ± 1.6 | 26 | 23 | mode of motion: multijoint exercises and single-joint exercises  exercise time: 75 mins  exercise frequency: three times per week | Mode of motion: daily activities | 24 weeks | 24 weeks | 3,4 |
| Staiano et al.[13] | 2013 | America | Overweight/Obese | 15-19 | 15-19 | 22 | 11 | mode of motion: exergame  exercise time: 30-60 mins  exercise frequency: per day | mode of motion: daily activities | 20 weeks | 20 weeks | 3 |
| Wagener et al.[14] | 2012 | America | Obese | 14 ± 1.66 | 14 ± 1.66 | 20 | 20 | mode of motion: exergame exercise  exercise time: 40 mins  exercise frequency: three times per week | mode of motion: daily activities | 10 weeks | 10 weeks | 1,2,3 |
| Watson et al.[15] | 2021 | Canada | Overweight/Obese girls | 14.8 ± 2.3 | 14.8 ± 2.3 | 47 | 7 | mode of motion: aerobic and resistance exercises  exercise time: 60-90 mins  exercise frequency: three times per week | mode of motion: daily activity | 12weeks | 12weeks | 4 |
| Weintraub et al.[16] | 2015 | USA | Overweight | 10-11 | 10-11 | 9 | 12 | mode of motion: aerobic exercise  exercise time: 75 mins  exercise frequency: three-four times per week | mode of motion: daily activities | 24 weeks | 24 weeks | 2,3 |
| Williams et al.[17] | 2019 | America | Overweight | 10.4 ± 0.87 | 10.4 ± 0.87 | 70 | 67 | mode of motion: aerobic activities and games  exercise time: 40 mins  exercise frequency: per day | mode of motion: daily activity | 32 weeks | 32 weeks | 2,4 |
| Young et al.[18] | 2004 | Korea | Obese | 10-12 | 10-12 | 34 | 28 | mode of motion: aerobic activities  exercise time: 60-70 mins  exercise frequency: per day | mode of motion: daily activities | 8 weeks | 8 weeks | 2 |
| Yu et al.[19] | 2020 | China | Obese | 9.9 ± 0.7 | 9.7 ± 0.6 | 99  (82/17) | 72  (54/18) | mode of motion: aerobic activities  exercise time: 60 mins  exercise frequency: five times per week | mode of motion: daily activities | 32weeks | 32weeks | 1,2 |

Notes:1 Anxiety, 2 Depression, 3 Self-esteem, 4 Self-worth

**References:**

1. CROKER H, VINER RM, NICHOLLS D, HAROUN D, CHADWICK P, EDWARDS C, WELLS J, WARDLE J: **Family-based behavioural treatment of childhood obesity in a UK national health service setting: randomized controlled trial**. *INT J OBESITY* 2012, **36**(1):16-26.

2. Daley AJ, Copeland RJ, Wright NP, Roalfe A, Wales JK: **Exercise therapy as a treatment for psychopathologic conditions in obese and morbidly obese adolescents: a randomized, controlled trial**. *PEDIATRICS* 2006, **118**(5):2126-2134.

3. Danielsen YS, Nordhus IH, Juliusson PB, Maehle M, Pallesen S: **Effect of a family-based cognitive behavioural intervention on body mass index, self-esteem and symptoms of depression in children with obesity (aged 7-13): a randomised waiting list controlled trial**. *OBES RES CLIN PRACT* 2013, **7**(2):e116-e128.

4. DeBar LL, Stevens VJ, Perrin N, Wu P, Pearson J, Yarborough BJ, Dickerson J, Lynch F: **A primary care-based, multicomponent lifestyle intervention for overweight adolescent females**. *Pediatrics (Evanston)* 2012, **129**(3):e611.

5. Goldfield GS, Kenny GP, Alberga AS, Prud'Homme D, Hadjiyannakis S, Gougeon R, Phillips P, Tulloch H, Malcolm J, Doucette S *et al*: **Effects of aerobic training, resistance training, or both on psychological health in adolescents with obesity: The HEARTY randomized controlled trial.** *J CONSULT CLIN PSYCH* 2015, **83**(6):1123-1135.

6. Heidarianpour A, Shokri E, Sadeghian E, Cheraghi F, Razavi Z: **Combined training in addition to cortisol reduction can improve the mental health of girls with precocious puberty and obesity**. *FRONT PEDIATR* 2023, **11**.

7. Lee G, Choi Y: **Effects of an obesity management mentoring program for Korean children**. *APPL NURS RES* 2016, **31**:160-164.

8. Lofrano-Prado MC, Donato Junior J, Lambertucci AC, Lambertucci RH, Malik N, Ritti-Dias RM, Correia MA, Botero JP, Prado WL: **Recreational Physical Activity Improves Adherence and Dropout in a Non-Intensive Behavioral Intervention for Adolescents With Obesity**. *RES Q EXERCISE SPORT* 2022, **93**(4):659-669.

9. Migueles JH, Cadenas-Sanchez C, Lubans DR, Henriksson P, Torres-Lopez LV, Rodriguez-Ayllon M, Plaza-Florido A, Gil-Cosano JJ, Henriksson H, Escolano-Margarit MV *et al*: **Effects of an Exercise Program on Cardiometabolic and Mental Health in Children With Overweight or Obesity: A Secondary Analysis of a Randomized Clinical Trial**. *JAMA NETW OPEN* 2023, **6**(7):e2324839.

10. Petty KH, Davis CL, Tkacz J, Young-Hyman D, Waller JL: **Exercise Effects on Depressive Symptoms and Self-Worth in Overweight Children: A Randomized Controlled Trial**. *J PEDIATR PSYCHOL* 2009, **34**(9):929-939.

11. Romero-Pérez EM, González-Bernal JJ, Soto-Cámara R, González-Santos J, Tánori-Tapia JM, Rodríguez-Fernández P, Jiménez-Barrios M, Márquez S, de Paz JA: **Influence of a Physical Exercise Program in the Anxiety and Depression in Children with Obesity**. *International Journal of Environmental Research and Public Health* 2020, **17**(13):4655.

12. Schranz N, Tomkinson G, Parletta N, Petkov J, Olds T: **Can resistance training change the strength, body composition and self-concept of overweight and obese adolescent males? A randomised controlled trial**. *BRIT J SPORT MED* 2014, **48**(20):1482-1488.

13. Staiano AE, Abraham AA, Calvert SL: **Adolescent exergame play for weight loss and psychosocial improvement: a controlled physical activity intervention**. *OBESITY* 2013, **21**(3):598-601.

14. Wagener TL, Fedele DA, Mignogna MR, Hester CN, Gillaspy SR: **Psychological effects of dance‐based group exergaming in obese adolescents**. *PEDIATR OBES* 2012, **7**(5).

15. Watson PM, McKinnon A, Santino N, Bassett-Gunter RL, Calleja M, Josse AR: **Integrating needs-supportive delivery into a laboratory-based randomised controlled trial for adolescent girls with overweight and obesity: Theoretical underpinning and 12-week psychological outcomes**. *J SPORT SCI* 2021, **39**(21):2434-2443.

16. Weintraub DL, Tirumalai EC, Haydel KF, Fujimoto M, Fulton JE, Robinson TN: **Team sports for overweight children: the Stanford Sports to Prevent Obesity Randomized Trial (SPORT)**. *Arch Pediatr Adolesc Med* 2008, **162**(3):232-237.

17. Williams CF, Bustamante EE, Waller JL, Davis CL: **Exercise effects on quality of life, mood, and self-worth in overweight children: the SMART randomized controlled trial**. *TRANSL BEHAV MED* 2019, **9**(3):451-459.

18. Moon YI, Park HR, Koo HY, Kim HS: **Effects of behavior modification on body image, depression and body fat in obese Korean elementary school children**. *YONSEI MED J* 2004, **45**(1):61.

19. Yu H, Li F, Hu Y, Li C, Yuan S, Song Y, Zheng M, Gong J, He Q: **Improving the Metabolic and Mental Health of Children with Obesity: A School-Based Nutrition Education and Physical Activity Intervention in Wuhan, China**. *NUTRIENTS* 2020, **12**(1):194.
