# Supplementary material for: Does physical activity really improve anxiety and depression in overweight or obese children and adolescents? A systematic review and meta-analysis
Source: BMC Psychiatry. 2026 Jan 16;26:139. doi: 10.1186/s12888-025-07761-9 (PMC12892821; doi:10.1186/s12888-025-07761-9)
Supplement: Supplementary file 1 — Supplementary Material 1 [file 12888_2025_7761_MOESM1_ESM.zip › Appendix/Additional file 9 Forest plot.docx]

Additional file 9 Forest plot

#### Forest plot for anxiety (n=5 studies)


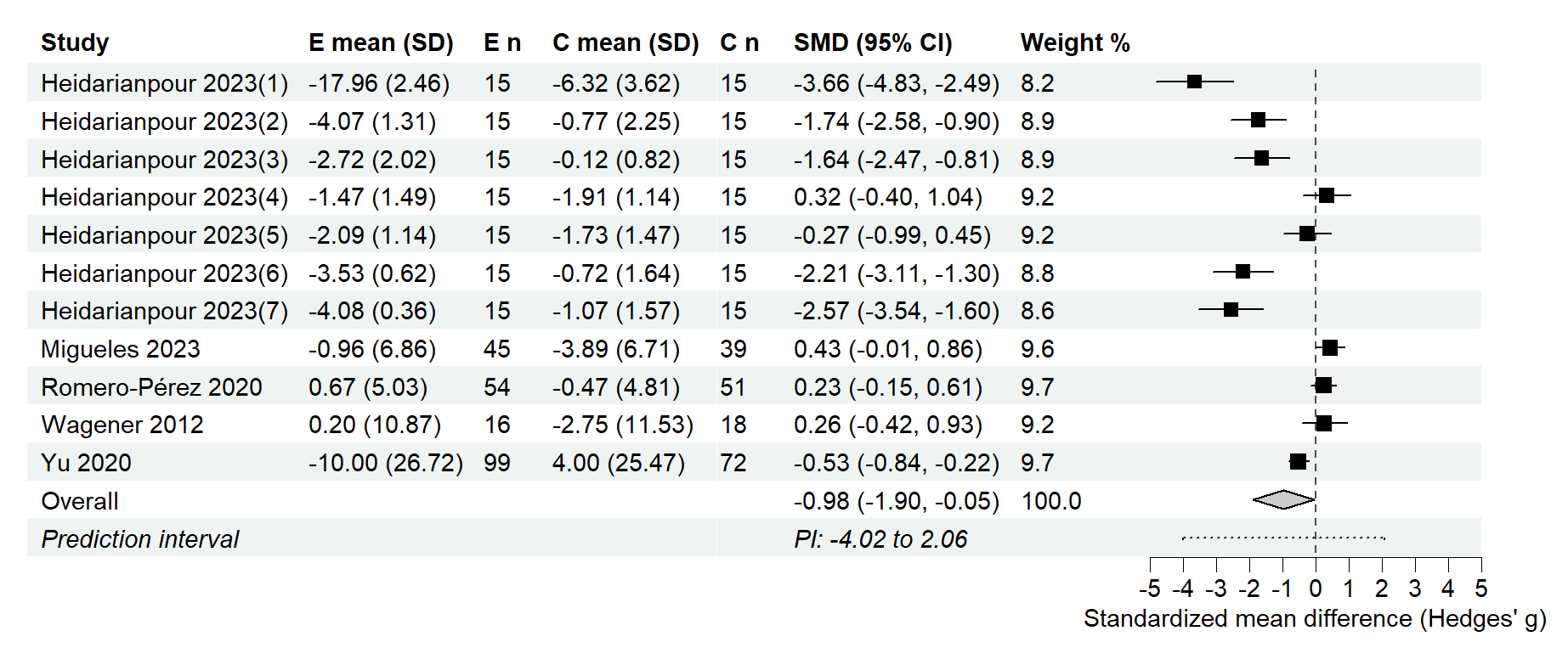


#### Forest plot for depression (n=14 studies)


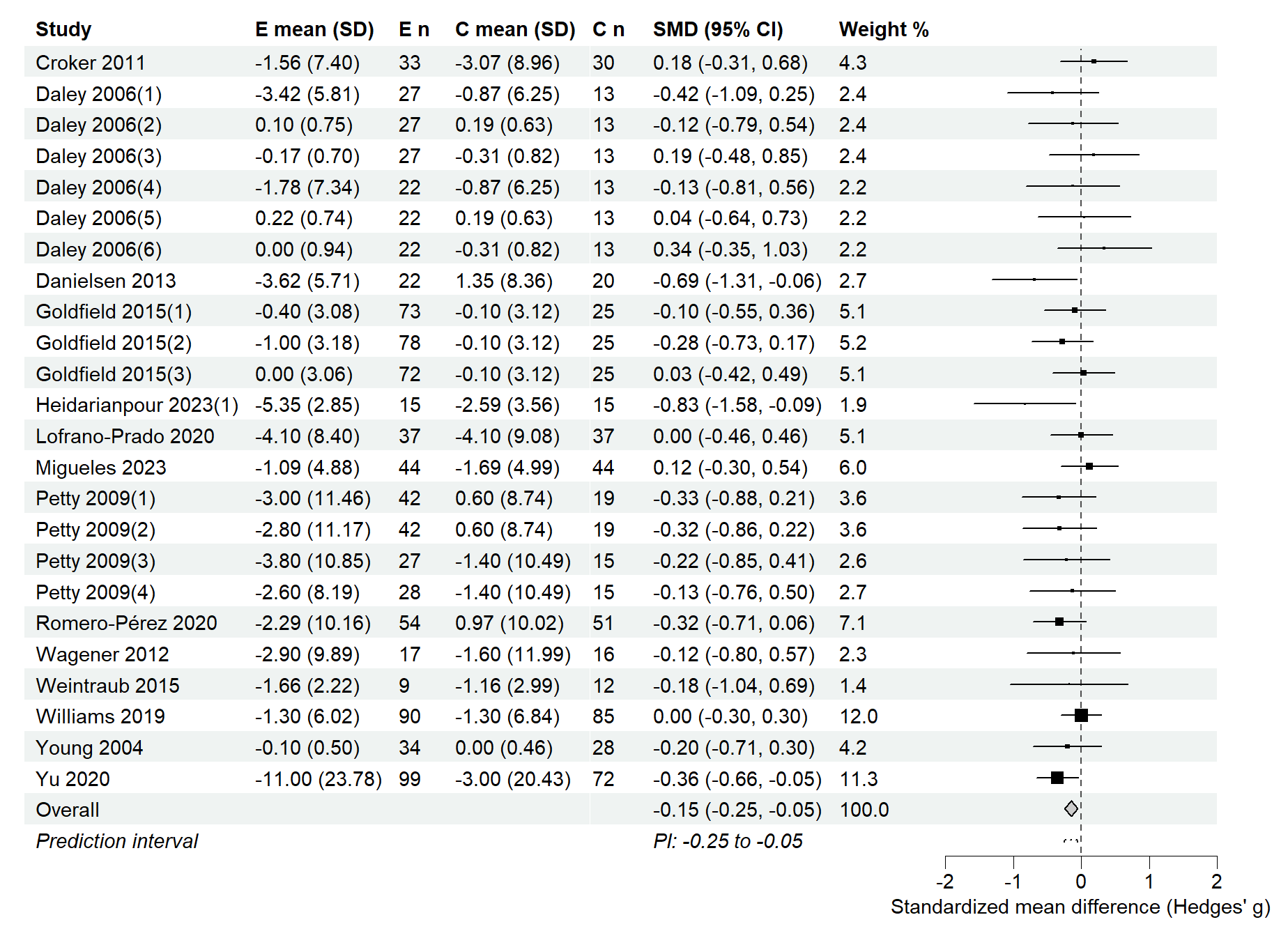


#### Forest plot for self-esteem (n=9 studies)


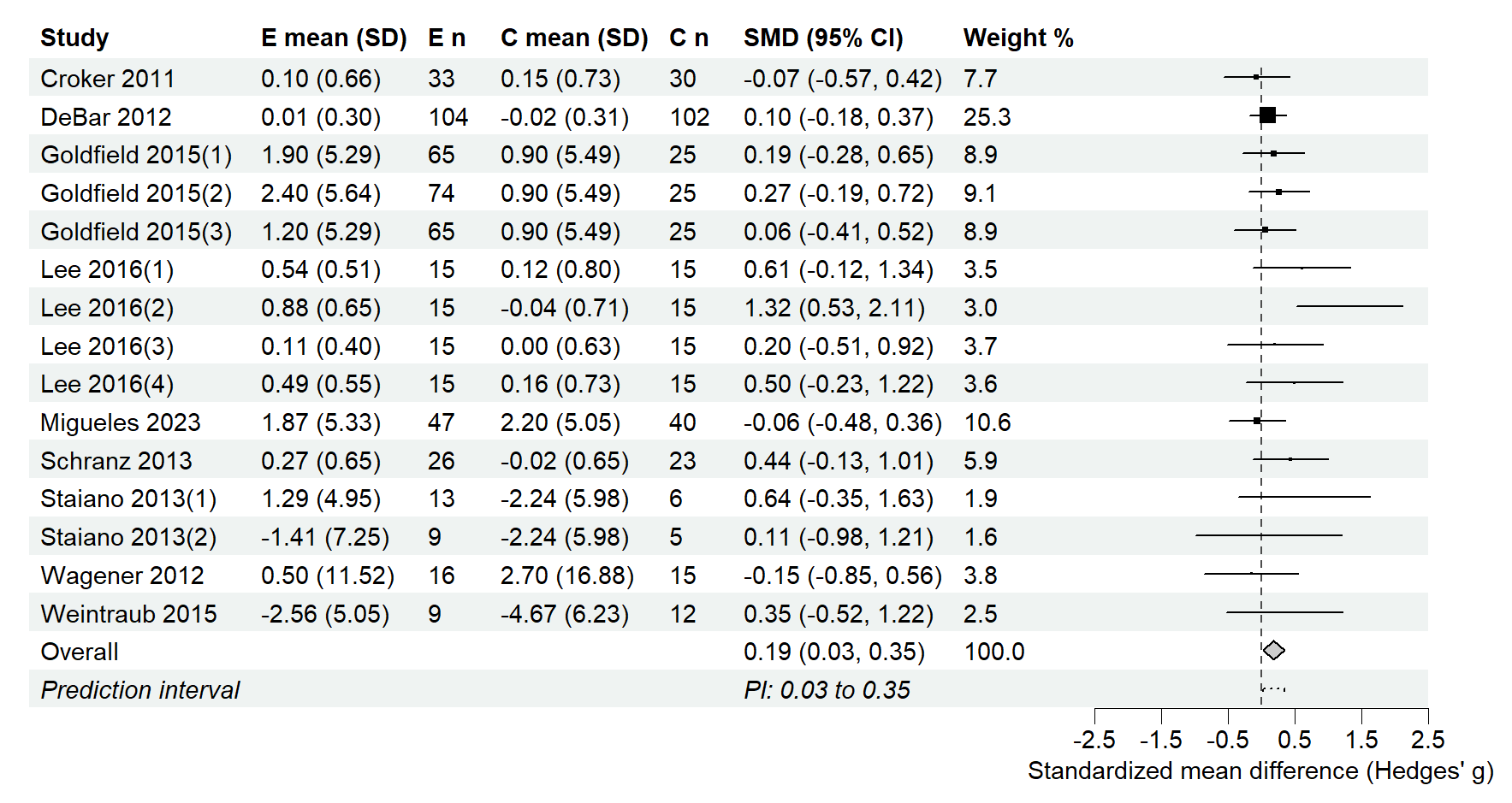


#### Forest plot for self-worth (n=7 studies)


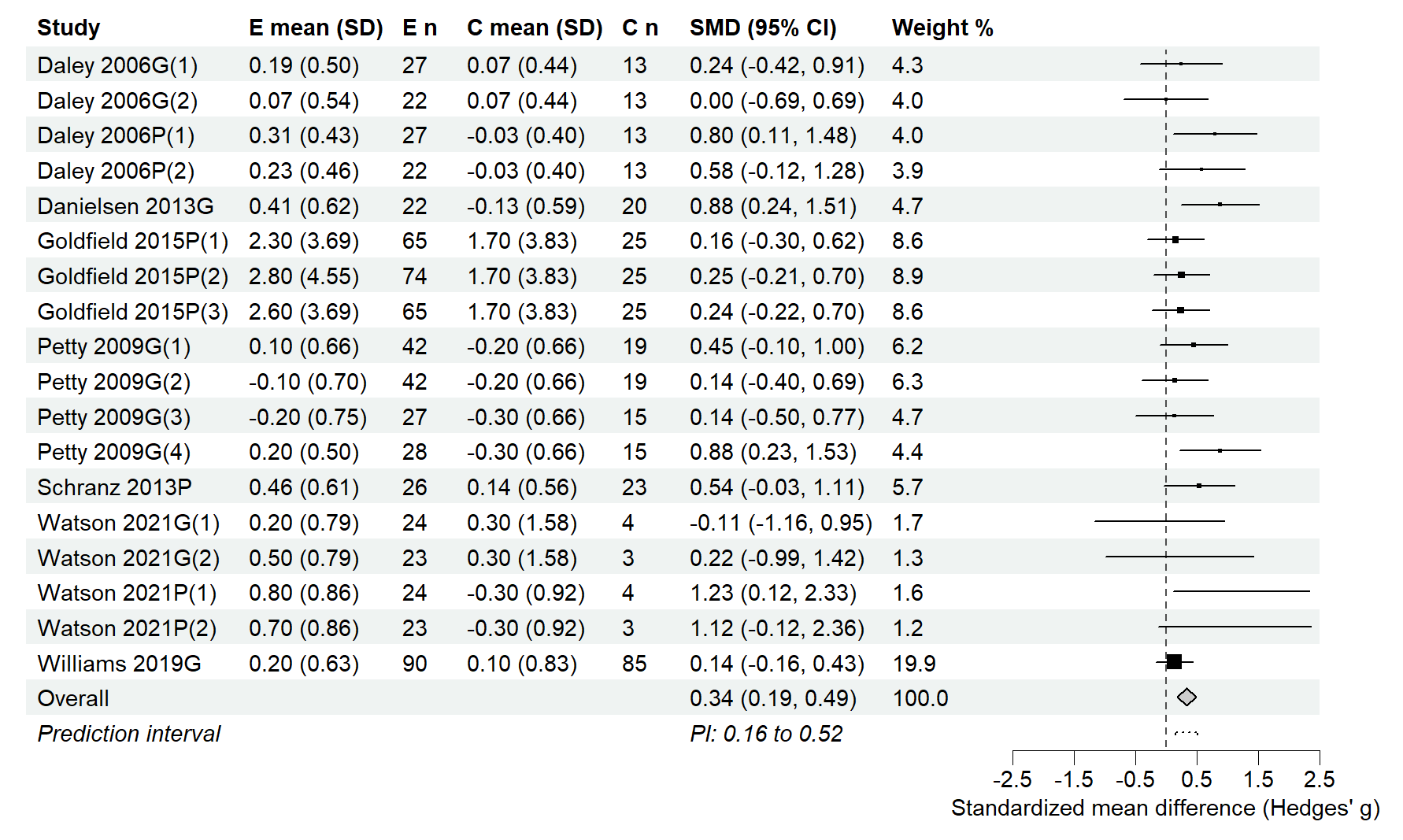


When a single study reported multiple intervention arms and/or multiple outcome subscales, each eligible comparison was included separately and labeled with numeric suffixes (e.g., Daley 2006(1-6)). Forest plot showing standardized mean differences (SMD, Hedges’ g) between the experimental (E) and control (C) groups across included studies. Values are presented as means with standard deviations and sample sizes for each group. Squares represent individual study effect estimates, with square size proportional to study weight under the random-effects model, and horizontal lines indicate 95% confidence intervals (CIs). The diamond represents the pooled effect estimate, with its width corresponding to the 95% CI. The vertical dashed line indicates the line of no effect (SMD = 0). The prediction interval (PI) reflects the range in which the true effect of a future study is expected to lie.
